# Selective activation of ABCA1/ApoA1 signaling in the V1 by magnetoelectric stimulation ameliorates depression via regulation of synaptic plasticity

**DOI:** 10.1016/j.isci.2022.104201

**Published:** 2022-04-04

**Authors:** Qingbo Lu, Fangfang Wu, Jiao Jiao, Le Xue, Ruize Song, Yachen Shi, Yan Kong, Jianfei Sun, Ning Gu, Ming-Hu Han, Zhijun Zhang

**Affiliations:** 1 Department of Neurology, Affiliated Zhongda Hospital, School of Medicine, Institution of Neuropsychiatry, Key Laboratory of Developmental Genes and Human Disease, Southeast University, Nanjing, Jiangsu 210096, China; 2 Department of Pharmacology, Nanjing University of Chinese Medicine, Nanjing, 210046, China; 3 Department of Biochemistry and Molecular Biology, School of Medicine, Southeast University, Nanjing, 210009, China; 4 State Key Laboratory of Bioelectronics, Jiangsu Key Laboratory of Biomaterials and Devices, School of Biological Science and Medical Engineering, Southeast University, Nanjing, 210009, China; 5 Department of Pharmacological Sciences, Department of Neuroscience, Center for Affective Neuroscience, and Friedman Brain Institute, Icahn School of Medicine at Mount Sinai, New York, NY 10029, USA; 6 Department of Mental Health and Public Health, Faculty of Life and Health Sciences, Shenzhen Institute of Advanced Technology, Chinese Academy of Sciences, Shenzhen, Guangdong 518055, China; 7 Department of Neurology, Affiliated Zhongda Hospital, School of Medicine, Institution of Neuropsychiatry, Key Laboratory of Developmental Genes and Human Disease, Southeast University, Nanjing, Jiangsu 210096, China; 8 Department of Mental Health and Public Health, Faculty of Life and Health Sciences, Shenzhen Institute of Advanced Technology, Chinese Academy of Sciences, Shenzhen, Guangdong 518055, China; 9 Research Center for Brain Health, Pazhou Lab, Guangzhou, Guangdong 510330, China

**Keywords:** Molecular neuroscience, Cellular neuroscience, Techniques in neuroscience

## Abstract

Emerging evidence suggests that dysfunction of the visual cortex may be involved in major depressive disorder (MDD). However, the underlying mechanisms remain unclear. We previously established that combined magnetic stimulation system treatment (c-MSST) resulted in an antidepressant effect in mice. In the present study, we found that V1-targeted c-MSST induced significant antidepressant effects in chronic unpredictable mild stress (CUMS)- and lipopolysaccharide (LPS)-treated mice. Proteomic screening investigation and repeatable validation revealed that expression of the V1 neuronal ATP-binding cassette transporter A1 (ABCA1) and apolipoprotein A-1 (ApoA1) was downregulated in CUMS mice, an effect that was normalized by c-MSST. Neuron-specific knockdown of ABCA1 in V1 blocked c-MSST’s antidepressant effects. Mechanistically, CUMS reduced dendritic spine density and long-term plasticity in V1, and these deficits were reversed by c-MSST. V1-targeted c-MSST was found to induce rapid antidepressant effects that are mediated by alterations in synaptic plasticity via the ABCA1/ApoA1 signaling pathway in V1.

## Highlights

• c-MSST targeting the primary visual cortex induced antidepressant effects
• ABCA1/ApoA1 signaling contributed to c-MSST-mediated antidepressant actions
• Magnetic stimulation of primary visual cortex enhanced synaptic plasticity
• Circulating levels of ApoA1 were lower in patients with depression

## Graphical abstract

**Figure:**
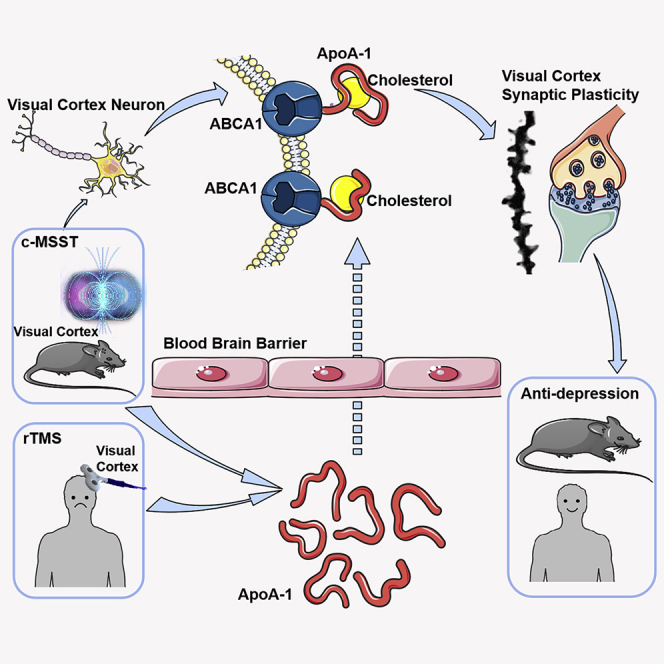

## Introduction

Major depressive disorder (MDD) is an extremely prevalent psychiatric illness that severely reduces the quality of life in patients, and this mental disorder is predicted to constitute the largest proportion of global disease burden by 2030 (Malhi and Mann, 2018). Although first-line drugs for the treatment of depression are available, they have several limitations such as delayed onset, low cure rate and limited efficacy (Zhu et al., 2020). Moreover, these limitations increase the rate of suicide, autotomy, and mental disability in MDD patients (McClintock et al., 2011). In 2008, the United States Food and Drug Administration approved the use of repetitive transcranial magnetic stimulation (rTMS) to treat adult treatment-resistant MDD (TRD) (Oberman et al., 2021), in which rTMS targets the left dorsolateral prefrontal cortex (DLPFC) at 10 Hz (George et al., 2000). Recently, our research team reported that individualized rTMS in the left visual cortex shows therapeutic effects without adverse reactions in drug-free MDD patients (Song et al., 2020; Zhang et al., 2021). However, investigating and understanding the neural mechanisms that underlie the effects of rTMS in the targeted brain regions in human subjects and animal models remain a challenge (Guadagnin et al., 2016; Meng et al., 2018). To address this issue, we developed a combined magnetic stimulation system (c-MSS) to achieve precise magnetic stimulation of the mouse left prelimbic (PrL) cortex (Lu et al., 2020), which was named c-MSS treatment (c-MSST). The mouse PrL cortex is potentially homologous to the DLPFC in primates (Vertes, 2006). In the present study, we examined whether c-MSST targeting the left primary visual cortex (V1) induces antidepressant effects in mouse models of depression and further explored the possible neurobiological mechanism.

Previous studies have revealed that structural and functional abnormalities of the visual cortex may be related to the pathogenesis of depression. For example, gamma-aminobutyric acid (GABA) levels (Kugaya et al., 2003; Price et al., 2009) and GABAergic neurons (Maciag et al., 2010) are remarkably decreased in the occipital cortex of MDD patients, which may relate to abnormalities in visual perception and psychopathological symptoms (Song et al., 2021). Compared with healthy controls, MDD patients exhibit reduced voxel-mirrored homotopic connectivity of the visual cortex, which is negatively associated with Hamilton Depression Rating Scale scores (Deng et al., 2021). Abnormalities in spontaneous activity have been observed in several brain regions in chronic unpredictable mild stress (CUMS) mice, including the visual cortex (Dong et al., 2020). Furthermore, reductions in responses to a flashing checkerboard stimulus measured by fMRI have been found in the visual cortex of depressed patients compared to healthy participants (Shaffer et al., 2018). Exposure to domestic violence during childhood is associated with reduced gray matter volume and thickness in the visual cortex of young adults (Tomoda et al., 2012), and such childhood trauma is closely linked to the pathophysiology of depression (Li et al., 2020). In addition, variation in the tumor necrosis factor-α (*TNF-α*) gene has been shown to selectively affect anatomy of the visual cortex in depressed subjects (Zhou et al., 2018). Functionally, the amplitudes of visually evoked potentials are significantly decreased in MDD patients compared to matched controls, and this deficit is restored by chronic antidepressant treatment (Normann et al., 2007). Ketamine is a rapid-acting antidepressant that results in faster GABA transmission in the early visual cortex of TRD patients (Gilbert et al., 2021). Based on the above findings, further investigations are needed to determine whether interventions involving the visual cortex or restoration of normal function in the visual cortex can improve depressive symptoms.

Numerous clinical and preclinical studies have also demonstrated that magnetoelectric therapy achieves antidepressant efficacy through regulation of structural and functional synaptic plasticity in various other brain regions (Murrough et al., 2017). High frequency rTMS induces phosphorylation of the neuronal ribosomal protein S6 in the piriform cortex, which may contribute to neuronal growth and synaptic plasticity (Fujiki et al., 2020). In contrast, low frequency magnetic stimulation influences regulation of structural synaptic plasticity in hippocampal neurons via activation of brain-derived neurotrophic factor (BDNF)-tropomyosin-related kinase B (TrkB) signaling pathways (Ma et al., 2013). Taken together, these findings suggest that synaptic plasticity plays an important role in mediating the benefits of magnetic therapy. Previous studies have reported that synaptic plasticity also exists in the visual cortex. For example, transcranial direct current stimulation (tDCS) induces long-term potentiation (LTP)-like plasticity in the visual cortex of healthy individuals (Frase et al., 2021). The first-line antidepressant fluoxetine has been shown to restore visual cortex plasticity in adult rats (Maya Vetencourt et al., 2008). However, it is unclear if MDD patients have synaptic plasticity dysfunction and if magnetoelectric stimulation can affect the synaptic plasticity of the visual cortex. Previously, we demonstrated that the biological effects of c-MSST were mainly regulated by neuronal activation of magneto-electric induction, rather than by thermal or vibration effects (Lu et al., 2020). Based on these findings, we hypothesized that c-MSST may improve depression-like behaviors by enhancing synaptic plasticity in the visual cortex.

In the present study, we investigated whether depression-like behaviors in mice could be ameliorated by c-MSST stimulating the V1. Utilizing quantitative proteomics and repeatable validation methods, we then explored the effects of c-MSST on various proteins and related signaling pathways in the V1. We also examined key proteins and developed viral tools for their manipulation. Finally, through viral manipulation, we explored the relationships between these proteins, c-MSST-induced antidepressant effects, and synaptic plasticity.

## Results

### Stability, safety and accuracy of c-MSST

**Figure 1:**
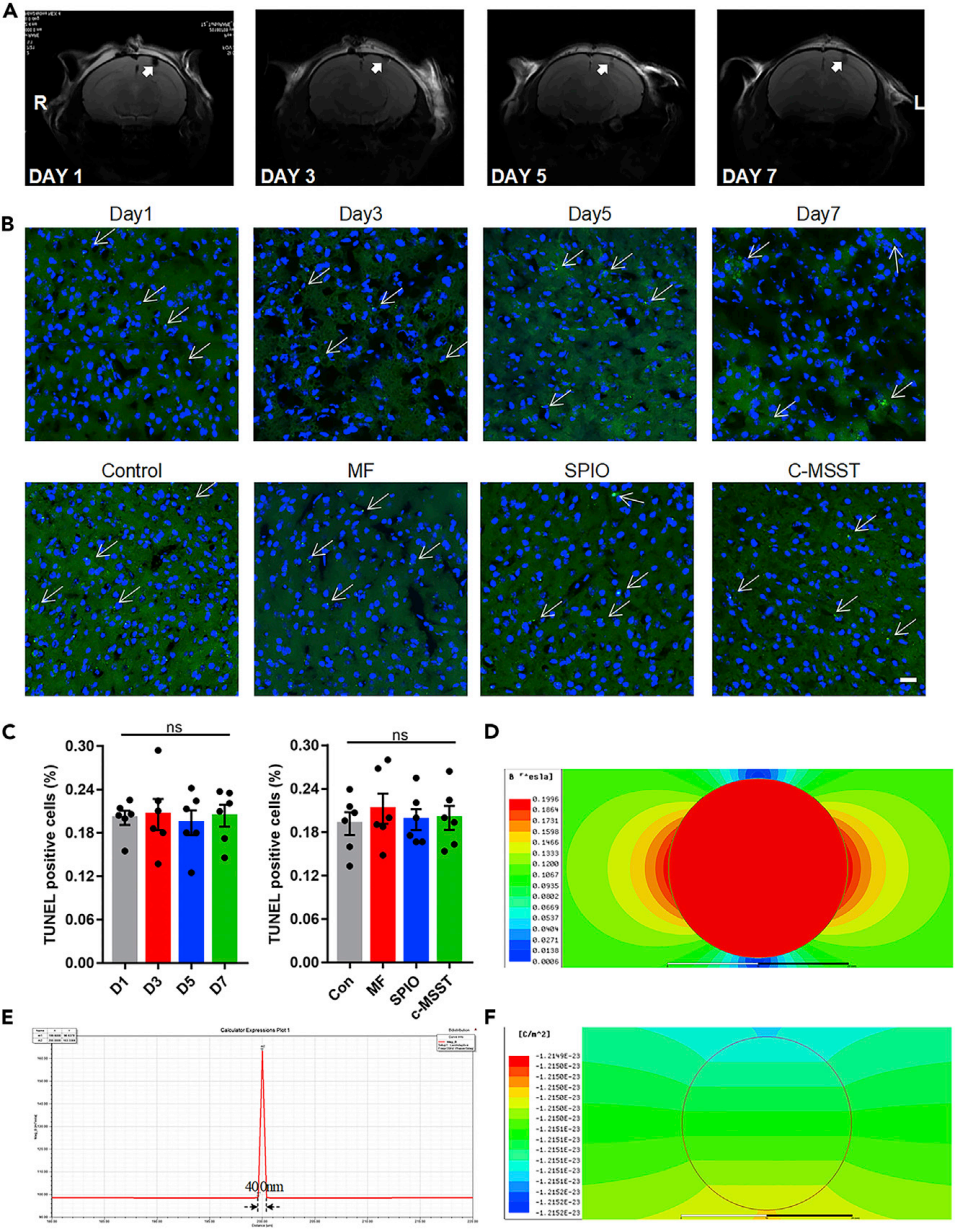
The evaluation of localization, safety and regional magnetoelectric effect of SPIO nanoparticles in the left V1 of mice (A) Representative T2-weighted MRI images of SPIO nanoparticles at 1, 3, 5, and 7 days after microinjection into the left V1 in mice. n = 6 mice per group. (B) TUNEL staining was performed to visualize apoptotic cells in the left V1 at 1, 3, 5 and 7 days after microinjection. Apoptotic cells were detected in the control group, MF group, SPIO group and c-MSST group at day 5 after microinjection. TUNEL (green)/DAPI (blue) double stained images are shown for each group. Scale bar = 50 μm. White arrows represent double-positive cells. (C) Quantification of TUNEL staining of mouse left V1. There were no significant differences at 1, 3, 5, and 7 days. There were no significant differences between sham-treated groups (MF and SPIO) and c-MSST group in five days. n = 6 mice per group. Data are expressed as mean ±SEM. p > 0.05 using one-way ANOVA with Bonferroni correction. (D) Magnetization simulation of a 20nm iron oxide nanoparticle under the stimulation of a 0.1 T pulsed magnetic field. The simulation was done by ANSYS 14.0 software and all the parameters, including the magnetic field for stimulation, the material composition and the ambient electromagnetic properties were set identical with the experimental cases. Along the direction of magnetic stimulation, the magnetic effect was seen to get greatly enhanced. (E) The quantitative calculation of the magnetic effect range for the simulation in Figure(D) It was shown that the maximal spreading range for a 20 nm iron oxide nanoparticles was about 400 nm. Actually, the effective range must be less than 400nm because zero field is absolutely unable to activate the cortex. (F) Calculated result of the electric displacement vector for the simulation in Figure(D) The electric displacement vector is production of the permittivity and the induced electric field intensity. According to the simulated result, the intensity of induced electric field was about 1.7 × 10-15V/m, and the induced electric field was highly localized around the nanoparticle surface. SPIO, superparamagnetic iron oxide; MRI, magnetic resonance imaging; TUNEL, terminal deoxynucleotidyl transferase-dUTP nick end labeling; MF, magnetic field; SPIO, superparamagnetic iron oxide; c-MSST, combined magnetic stimulation system treatment; ns, not significant; DAPI,4′,6-diamidino-2-phenylindole.

Following microinjection into the left V1 (Hammad et al., 2015) of control mice, superparamagnetic iron oxide (SPIO) nanoparticles were visible as sphere-like dark regions on magnetic resonance imaging (MRI) and remained stable within the V1 for at least 7 days (Figure 1A). Even 7 days after microinjection, SPIO nanoparticles were not toxic to cells within the V1 of mice, as evidenced by terminal deoxynucleotidyl transferase-dUTP nick end labeling (TUNEL) assay (Figures 1B and 1C). Consistently, cell apoptosis in the left V1 was not affected by magnetic field (MF), SPIO nanoparticles, or c-MSST compared with control mice after 5 days (Figures 1B and 1C). Of importance, as shown in Figure 1D, the field of magnetization along the diameter of magnetic nanoparticles was significantly enhanced and highly localized. Figure 1E shows that the maximal range was about 400 nm for a 20 nm particle. Furthermore, the electric flux density induced by the alternating magnetic field was effective just around the surface of the nanoparticles (Figure 1F). The enhanced magnetic effect was only significant very near the nanoparticles and was free of distant spread across the cortices. In sum, the above findings suggest that the accurate action of the c-MSST was focused on the SPIO nanoparticles injected in the V1 region.

### Effects of c-MSST targeting the V1 on depression-like behaviors in chronic unpredictable mild stress (CUMS)- and lipopolysaccharide (LPS)-treated mice

After CUMS treatment for 5 weeks, depression-like behaviors were confirmed by lower sucrose consumption in sucrose preference test (SPT) (Figure S1A), longer immobility time in the forced swim test (FST) and tail suspension test (TST) (Figures S1B and S1C) compared to baseline data. Moreover, successful establishment of CUMS mice was ascertained by a significant decrease in body weight (Figure S1D) alongside a dramatic increase in coat scores (Figure S1E).

To optimize the stimulation frequency used, we followed the experimental procedure shown in Figure S2A. We examined therapeutic effects of c-MSST targeting the V1 on depression-like behaviors. Behavioral tests showed that both 5 and 10 Hz c-MSST for 5 days significantly ameliorated depression-like behaviors in CUMS mice, as indicated by increased sucrose consumption in SPT and reduced immobility time in FST and TST compared with CUMS group (Figures S2B andS2D). c-MSST had no effect on the distance traveled or time spent in central zone (as measures of anxiety-like behaviors) between groups (Figures S2E and S2F). Of interest, c-MSST only with 5 Hz stimulation for 5 days normalized the averaged distance and velocity in CUMS mice during the OFT (Figures S2G and S2H). To select optimal treatment time, we designed tests as illustrated in Figure S2I. Significant improvements in depression-like behaviors were observed after 5 days of c-MSST, and the therapeutic effects remained stable at day 7 (Figures S2J–S2P). Therefore, 5 Hz c-MSST intervention for 5 days was used in the subsequent experiments in this study.

**Figure 2:**
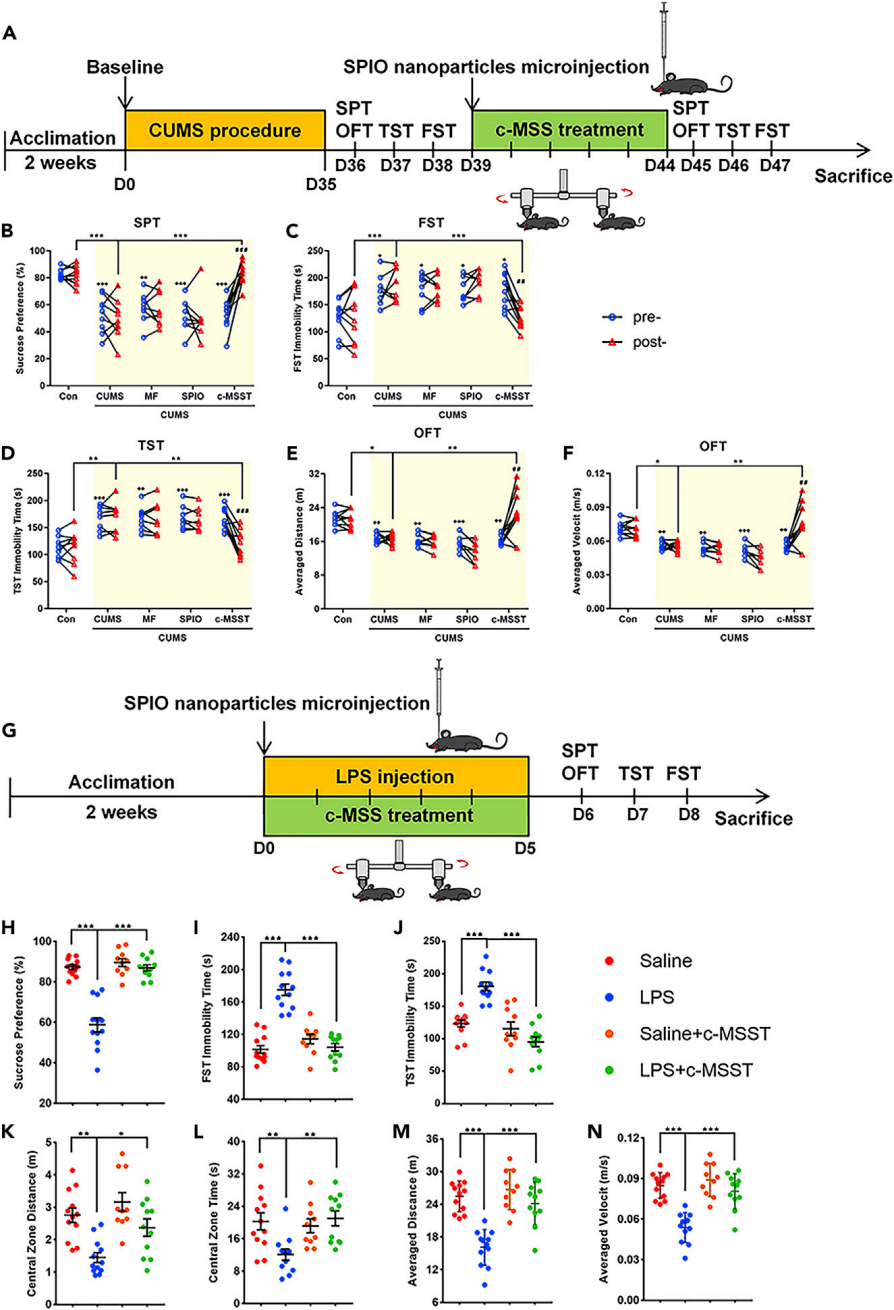
c-MSST targeting the left V1 prevents CUMS- and LPS-induced depression-like behaviors in mice (A) Schematic diagram of the experimental procedure for CUMS and c-MSST. (B–D) 5-Hz c-MSST targeting the V1 for 5 days induced observable antidepressant effects in CUMS mice confirmed by the SPT, FST, and TST behavioral tests. (E and F) 5-Hz c-MSST targeting the V1 for 5 days observably improved spontaneous locomotor activity in CUMS mice tested by the OFT. n = 8–10 mice per group. Data are expressed as the mean ±SEM. ^##^p < 0.01, ^###^p < 0.01 versus prec-MSST; ^+^ p < 0.05, ^++^ p < 0.01, ^+++^p < 0.001 versus Con prec-MSST; ∗p < 0.05, ∗∗p < 0.01, ∗∗∗p < 0.001 using ANOVA with Sidak correction. (G) Schematic diagram of the experimental procedure for LPS and c-MSST. (H–J) 5-Hz c-MSST targeting the V1 for 5 days induced a rapid antidepressant effects in LPS mice confirmed by the SPT, FST and TST behavioral tests. (K–N) 5-Hz c-MSST targeting the V1 for 5 days significantly improved anxiety-like behaviors and spontaneous locomotor activity in LPS mice. n = 10-12 mice per group. Data are expressed as mean ±SEM. ∗p < 0.05, ∗∗p < 0.01, ∗∗∗p < 0.001 using ANOVA with Bonferroni correction. SPIO, superparamagnetic iron oxide; MF, magnetic field; c-MSST, combined magnetic stimulation system treatment; CUMS, chronic unpredictable mild stress; LPS, lipopolysaccharide; SPT, sucrose preference test; FST, forced swim test; TST, tail suspension test; OFT, open field test. See also Figures S1 and S2.

To further verify therapeutic effects of c-MSST on CUMS-induced depression-like behaviors, an equivalent volume of saline was microinjected into the left V1 under magnetic stimulation conditions, or microinjection of SPIO nanoparticles into the left V1 was performed in absence of MF circumstances as controls. The study design schedule is depicted in Figure 2A. As shown in Figures 2B–2F, CUMS procedure decreased sucrose preference in SPT, increased immobility time in FST and TST and reduced the averaged distance and velocity compared with control group. These deficits in CUMS mice were largely rescued by 5 Hz c-MSST for 5 days, but not by control treatments (Figures 2B–2F). Overall, these results support that c-MSST induced observable antidepressant effects in CUMS mice.

To further confirm these findings, the same tests were performed in mice treated with intraperitoneal LPS injection for 5 days (Figure 2G). As expected, findings of 5 Hz c-MSST for 5 days in LPS model of depression were similar to those in CUMS model (Figures 2H–2J). In addition, LPS treatment induced anxiety-like behaviors that were significantly improved after c-MSST (Figures 2H–2N). Taken together, these behavioral data provide consistent evidence that c-MSST induces observable antidepressant effects in different mouse models of depression.

### Proteomics screening and validation of key proteins in the V1 of CUMS mice

**Figure 3:**
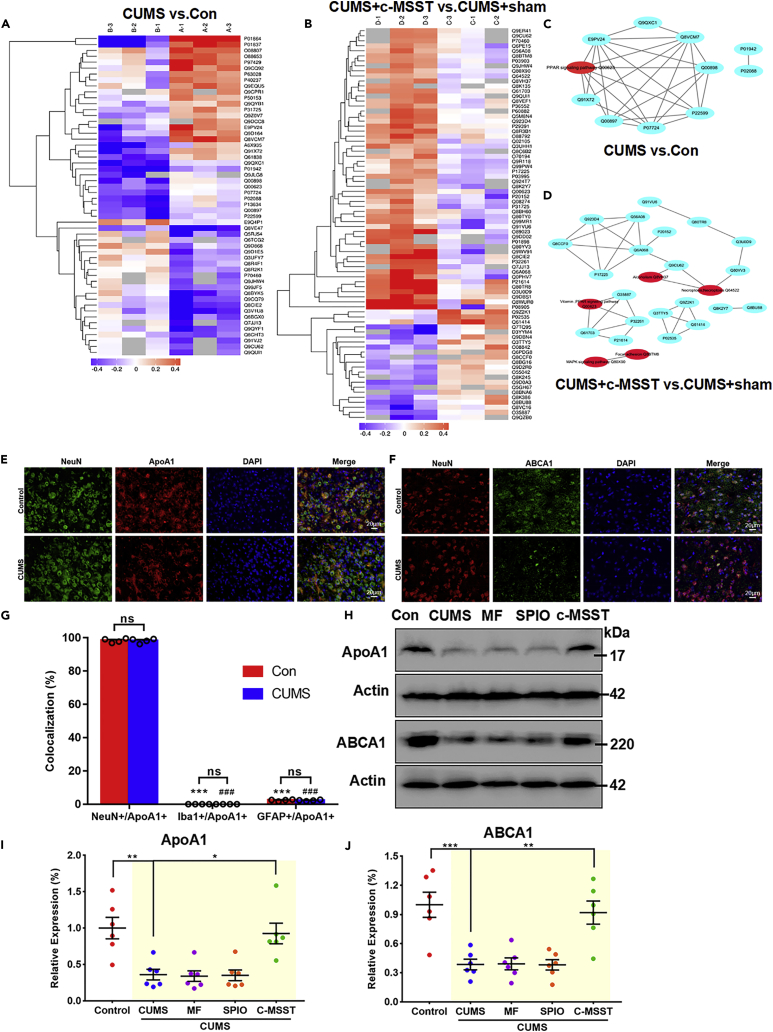
Activated ABAC1/ApoA1 signaling is involved in the antidepressant action of c-MSST targeting the left V1 of CUMS mice (A and B) Heat map of the differentially expressed proteins analyzed by iTRAQ (Group A, Control mice; Group B, CUMS mice; Group C, Sham c-MSST mice; Group D, c-MSST mice), n = 3 mice per group. (C and D) PPI network of the differential proteins in the four groups. The red circles indicate the major/hub nodes of the PPI network. (E and F) Representative fluorescence images of the mouse V1. Immunofluorescent double labeling for ApoA1 (red)/NeuN (green) and ABCA1 (green)/NeuN (red). Nuclei were stained blue with DAPI. n = 4 mice per group. Scale bar, 20 μm. (G) Colocalization of the three neurocytemarkers with ApoA1 was quantified in the left V1 of control and CUMS mice. n = 8–9 mice per group. Data are expressed as the mean ±SEM. ∗∗∗p < 0.001 versus Con-NeuN+/ApoA1+; ^###^p < 0.001 versus CUMS-NeuN+/ApoA1+ using ANOVA with Sidak correction. (H–J) Expression of ApoA1 and ABCA1 in the left V1 of control and CUMS mice. n = 6 mice per group. Data are expressed as mean ±SEM. ∗p < 0.05, ∗∗p < 0.01, ∗∗∗p < 0.001 using ANOVA with Bonferroni correction. iTRAQ, isobaric tagging for relative and absolute quantification; PPI, protein-protein interaction; ApoA1, apolipoprotein A1; ABCA1, ATP-binding cassette, subfamily A, member one; NeuN, neuronal nuclei; DAPI, 4′,6-diamidino-2-phenylindole; SPIO, superparamagnetic iron oxide; MF, magnetic field; c-MSST, combined magnetic stimulation system treatment; CUMS, chronic unpredictable mild stress; ns, non-significant. See also Figures S3 and S4.

**Figure 4:**
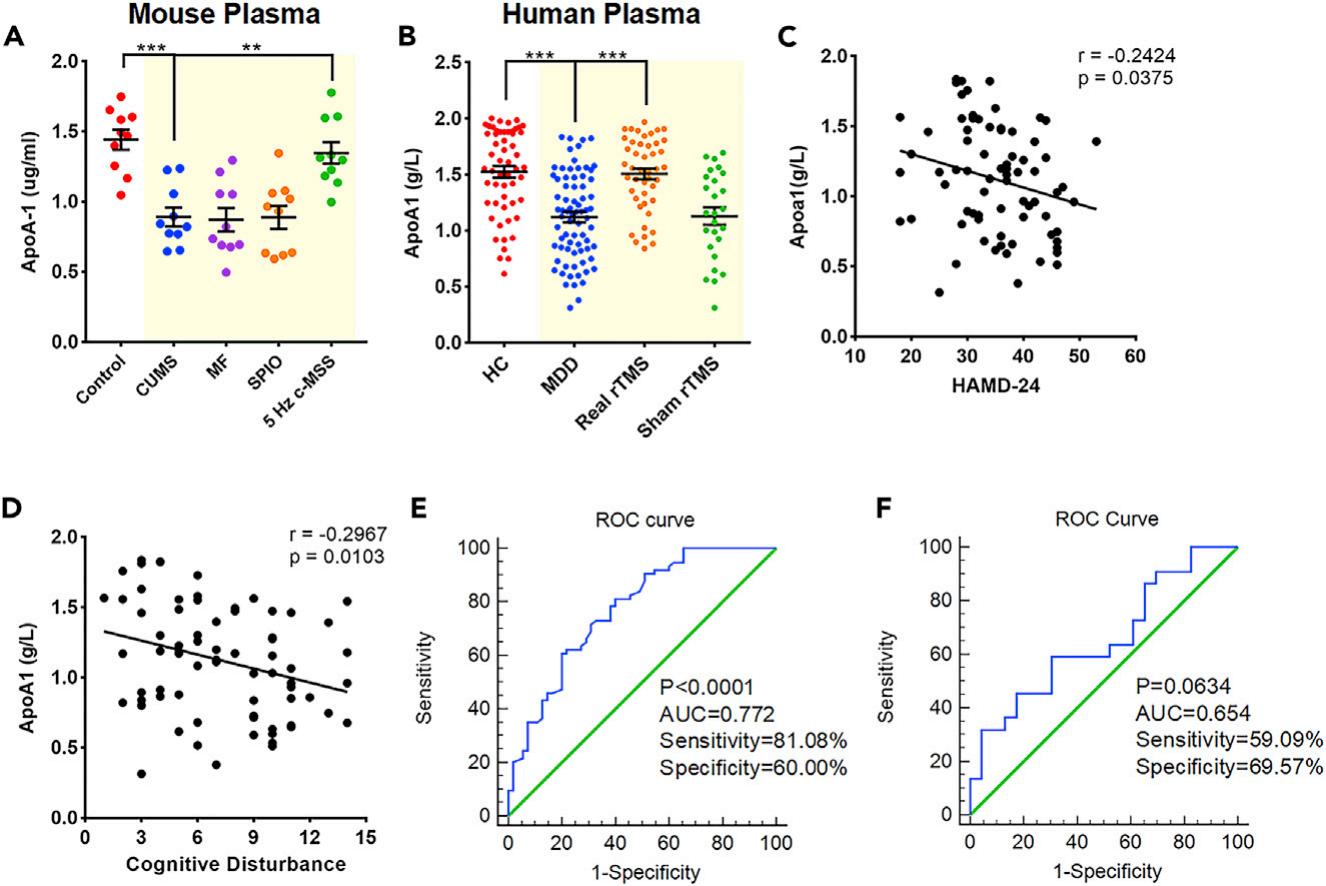
Circulating ApoA1 levels in MDD patients and mice with depression-like behaviors (A) Plasma levels of ApoA1 in CUMS mice, sham-treated mice and c-MSST mice. n = 10 mice per group. (B) Plasma levels of ApoA1 in HC (n = 55), MDD patients at baseline (n = 74) and after real (n = 45)/sham (n = 26) visual cortex rTMS treatment for 5 days. Data are expressed as the mean ±SEM. ∗∗p < 0.01, ∗∗∗p < 0.001 using ANOVA with Bonferroni correction. (C) Negative correlations between ApoA1 and HAMD-24 in MDD patients at baseline. (D) Negative correlations between ApoA1 and cognitive disturbance factor (HAMD). (E) ROC curve for the diagnostic performance of plasma ApoA1. (F) ROC curve of plasma ApoA1 for predicting the response to 5 days real rTMS treatment. ApoA1, apolipoprotein A1; CUMS, chronic unpredictable mild stress; superparamagnetic iron oxide; MF, magnetic field; c-MSST, combined magnetic stimulation system treatment; HC, healthy control; MDD, major depressive disorder; rTMS, repetitive transcranial magnetic stimulation; HAMD-24, 24-item Hamilton Depression Rating Scale. See also Tables S1 and S2.

To probe the molecular mechanisms underlying the antidepressant effects of c-MSST, we collected left V1 tissues from mice for proteomic analysis and probed potential molecular targets. The isobaric tagging for relative and absolute quantification (iTRAQ)-based relative quantitative proteomics analysis identified differentially expressed proteins in the V1 of four groups of mice (Figures 3A and 3B) with a change greater than 1.2-fold (up-regulation greater than 1.2-fold or down-regulation less than 0.83-fold, with p < 0.05 indicating differential proteins). Compared with control mice, 54 proteins were differentially altered in CUMS mice (23 up-regulated and 31 down-regulated). There were 56 up-regulated and 22 down-regulated proteins in c-MSST CUMS mice compared with sham-treated CUMS mice (Figures 3A and 3B). Notably, protein-protein interaction (PPI) analysis demonstrated that apolipoprotein A1 (ApoA1) protein in the left V1 decreased most significantly after exposure to CUMS, but was rescued after c-MSST (Figures 3C and 3D). The circulating levels of ApoA1 were also decreased in CUMS mice, an observation that was restored by c-MSST (Figure 4A). We further examined the plasma levels of ApoA1 in both MDD patients. The sociodemographic and clinical characteristics of normal controls and depressive subjects were listed in Tables S1 and S2. Compared with healthy control subjects, the plasma levels of ApoA1 were declined in MDD patients, which were largely reversed by visual cortex r-TMS for 5 days (Figure 4B). Furthermore, we observed a negative correlation between ApoA1 levels and the scores of 24-item Hamilton Depression Rating Scale (HAMD-24) (Figure 4C). Similar results were also observed regarding the negative association of ApoA1 levels with cognitive disturbance factors in HAMD-24 (Figure 4D). The analysis of receiver-operating characteristic (ROC) curve disclosed that the changes in plasma ApoA1 levels were able to serve as a biomarker for depression in MDD patients (Figures 4E and 4F). Collectively, these results indicate that c-MSST mice may exhibit resistance to stress-induced development of depression-like behaviors via regulation of proteins positioned in ApoA1.

The immunofluorescence double staining results showed that ApoA1 was selectively co-expressed within neurons (Figures 3E and 3G), but not co-localized with microglia or astrocytes (Figures S3A and 3B). Notably, published findings have verified that cells in the CNS do not produce ApoA1 (Dergunov et al., 2017; Litvinov et al., 2016). ApoA1 is recognized as a primary mediator of cholesterol efflux from lipid-loaded macrophages via cholesterol transporter ATP-binding cassette, subfamily A, member 1 (ABCA1) (Dergunov et al., 2017; Litvinov et al., 2016). On these grounds, we assessed ABCA1 protein expression in the left V1, which originates from neurons, in both control and CUMS mice. Consistent with ApoA1 levels, expression of ABCA1 in the left V1 was remarkably downregulated in CUMS mice (Figure 3F). The immunoblotting results further demonstrated that the decreased protein expression of ABCA1 and ApoA1 was significantly restored after c-MSST (Figures 3H–3J). ABCA1 loaded the outflow of cholesterol and phospholipids from cells to lipid-poor apolipoproteins (ApoA1 and ApoE), thus generating nascent high-density lipoproteins (Dergunov et al., 2021). A significant increase in cholesterol intensity might disrupt synaptic plasticity and lead to cognitive impairment (Loera-Valencia et al., 2021). Given the importance of the interaction between ABCA1 and ApoA1 in cholesterol metabolism, we hypothesized that downregulation of ABCA1/ApoA1 might give rise to higher cholesterol contents in V1 of CUMS mice. Filipin staining results and cholesterol content indicated that an obvious decrease in cholesterol synthesis in the V1region of CUMS mice receiving c-MSST (Figure S4). Such effects of c-MSST were further confirmed by measurement of total cholesterol (Figure S4). These results suggested that deposition of cholesterol in the V1 because of the downregulation of ABCA1/ApoA1 could be attenuated by c-MSST.

### Effects of c-MSST targeting the V1 on ABCA1/ApoA1-mediated synaptic plasticity

**Figure 5:**
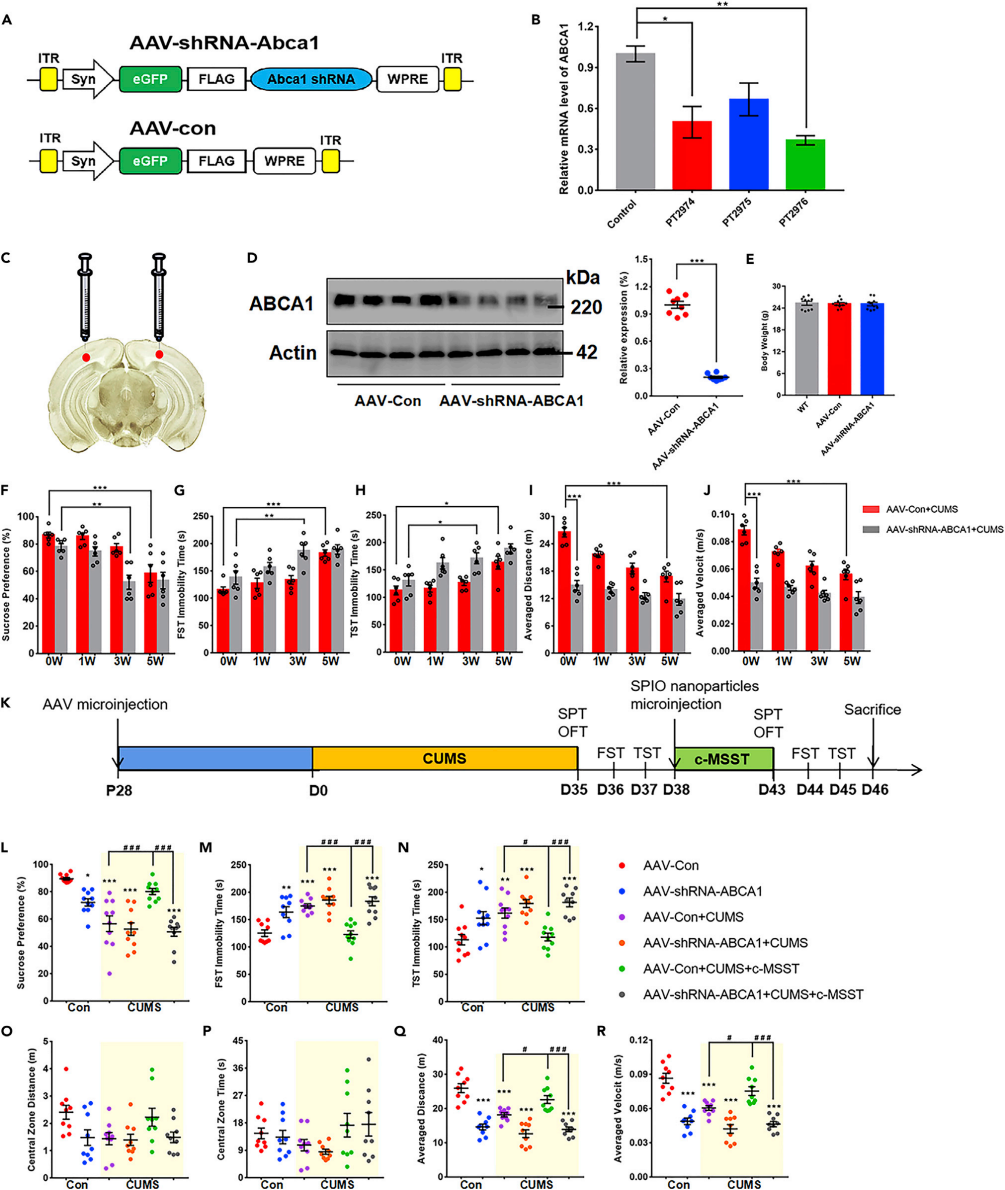
Selective downregulation of ABCA1 in neurons inhibits the antidepressant effects of c-MSST intervention targeting the V1 of CUMS mice (A) Schematics of the AAV constructs (AAV2/9 serotype) for neuron-specific knockdown of ABCA1 (AAV-shRNA-ABCA1) or the negative control (AAV-Con). (B) The sequences for shRNA-ABCA1 with the highest interference efficiency were confirmed by RT-PCR. n = 3 mice per group. Data are expressed as mean ±SEM. ∗p < 0.05, ∗∗p < 0.01 using ANOVA with Bonferroni correction. (C) Illustration of bilateral viral injections into the mouse V1. (D) Expression of ABCA1 in the V1 of mice with AAV-Con and AAV-shRNA-ABCA1 for 4 weeks n = 8 mice per group. ∗∗∗p < 0.001 versus AAV-Con group using Student’s t-test. (E) Body weight. n = 10 mice per group. (F–H) Depression-like behaviors in neuron-specific ABCA1 knockdown mice. (I and J) Spontaneous locomotor activities of neuron-specific ABCA1 knockdown mice. n = 6 mice per group. Data are expressed as mean ± SEM ∗p < 0.05, ∗∗p < 0.01, ∗∗∗p < 0.001 using ANOVA with Bonferroni correction. (K) Schematic diagram of the experimental procedure. (L–N) Depression-like behaviors after 5-Hz c-MSST for 5 days. (O–R) Anxiety-like behaviors and spontaneous locomotor activity after 5-Hz c-MSST for 5 days. n = 9–10 mice per group. Data are expressed as mean ±SEM. ∗p < 0.05, ∗∗p < 0.01, ∗∗∗p < 0.001 versus AAV-Con group, ^#^p < 0.05, ^##^p < 0.01, ^###^p < 0.001 using ANOVA with Bonferroni correction. AAV, adeno-associated viral; SPIO, superparamagnetic iron oxide; MF, magnetic field; c-MSST, combined magnetic stimulation system treatment; CUMS, chronic unpredictable mild stress; SPT, sucrose preference test; FST, forced swim test; TST, tail suspension test; OFT, open field test. See also Figure S5.

Next, we validated the specific actions of c-MSST on ABCA1/ApoA1 axis by microinjecting either adeno-associated virus (AAV)-carrying control short hairpin RNA (shRNA) or ABCA1 shRNA into the V1 of mice, as illustrated in Figures 5A and 5C. After AAV microinjection, enhanced green fluorescent protein (eGFP) was abundantly expressed in the left V1 of mice (Figure S5). Interference efficiency was examined after AAV transduction *in vivo*, and significant decreases in ABCA1 mRNA and protein expression were observed in ABCA1 shRNA-injected mice compared with controls (Figures 5B and 5D). Four weeks after AAV delivery, mice were subjected to the CUMS procedures for 5 weeks followed by behavioral testing (Figure 5K). Although there was no effect on body weight (Figure 5E), mice with neuron-specific ABCA1 knockdown displayed depression-like behaviors within 3 weeks, which is similar to the depression-like behaviors observed at 5 weeks in CUMS-treated control mice (Figures 5F–5H). Of interest, the mice exhibited lower spontaneous locomotor activity when neuronal ABCA1 in the V1 was deleted for 1 week, which was comparable to the level in mice that underwent the CUMS procedure for 5 weeks (Figures 5I and 5J). Although depression-like behaviors did not differ between the two groups of mice at the end of the CUMS protocol (Figures 5F–5J), the behavioral results described above strongly suggest that V1 neuronal ABCA1-deficient mice are more susceptible to CUMS, indicating that neuronal ABCA1 deficiency in the V1 may be an essential condition for development of depression under chronic stress conditions.

The process of examining whether neuronal specific ablation of ABCA1 abolishes the antidepressant effects of V1-targeted C-MSST is illustrated in the flow diagram in Figure 5K. As mentioned above, CUMS mice exhibited lower sucrose consumption in the SPT (Figure 5L), higher immobility time in both FST and TST (Figures 5M and 5N), and lower average distance and velocity (Figures 5Q and 5R), and these abnormalities were all normalized after c-MSST intervention in the V1. However, genetic depletion of ABCA1 almost completely eliminated the antidepressant effects of c-MSST (Figures 5L–5R). Consistent with the above results, travel distance and time spent in central zone did not differ significantly between mouse groups (Figures 5O and 5P). Altogether, our data support that ABCA1/ApoA1 signaling pathway in V1 neurons is necessary to mediate c-MSST-induced antidepressant effects.

**Figure 6:**
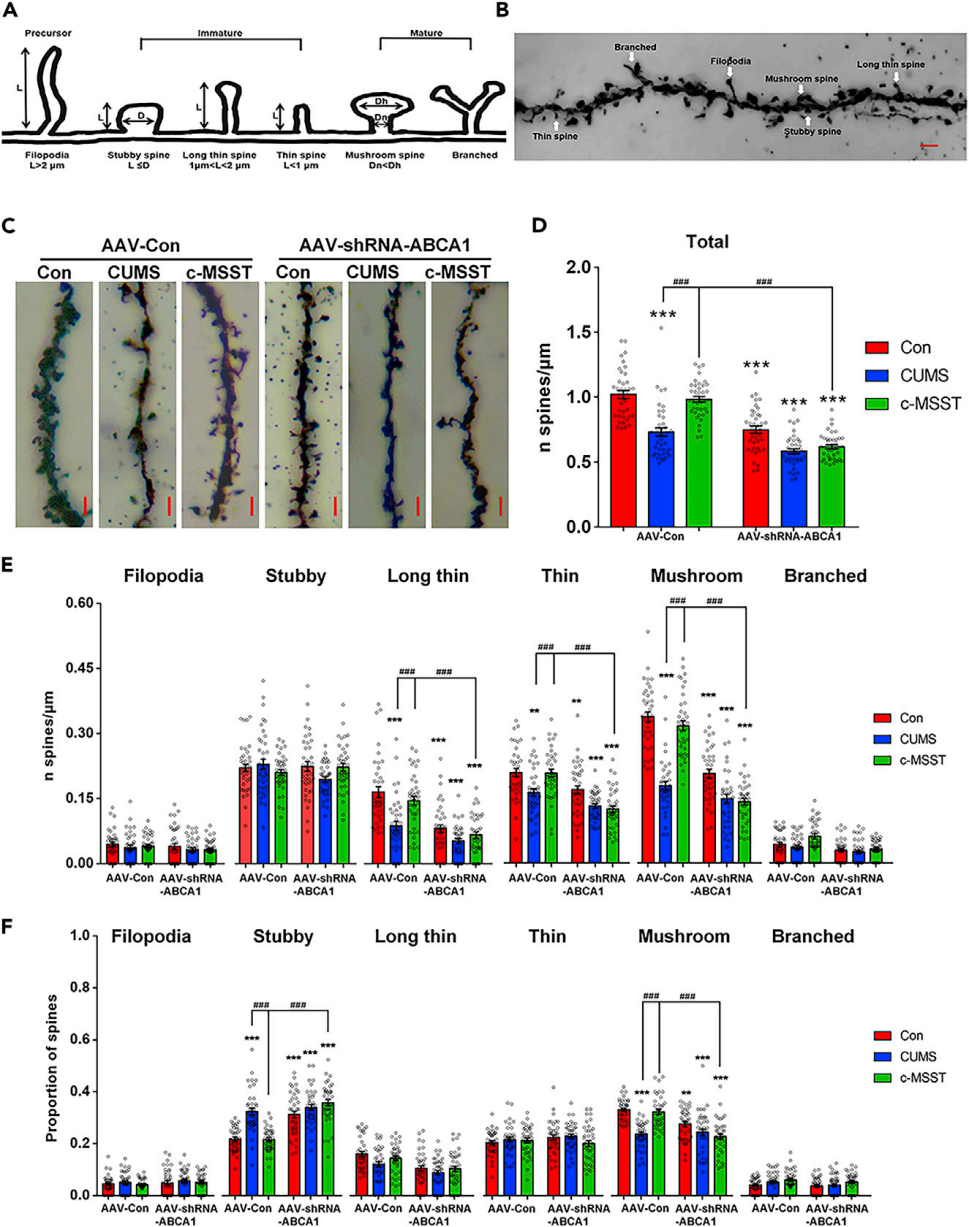
c-MSST exerts antidepressant effects via maintaining normal dendritic morphology and density (A) Diagram of dendritic spines with different morphologies. Filopodia spines are more than 2 μm in length; the maximum width of stubby spines is less than its length; long thin spines are 1–2 μm in length; thin spines are less than 1 μm in length; mushroom spines’ head/neck diameter ratio are more than 1. (B) Golgi-Cox stained dendritic spines in the mouse V1. Scale bar = 2 μm. (C–F) Representative image of spines and bar graph of spine density in the dendrites of V1 neurons. n = 38-40 neurons from five mice per group. Scale bar = 2 μm ∗p < 0.05, ∗∗p < 0.01, ∗∗∗p < 0.001 versus AAV-Con group, ^#^p < 0.05, ^##^p < 0.01, ^###^p < 0.001 using ANOVA with Bonferroni correction. CUMS, chronic unpredictable mild stress; c-MSST, combined magnetic stimulation system treatment.

Dendritic spines are tiny postsynaptic processes from dendrites that receive most excitatory synaptic inputs in the brain. Importantly, it is known that abnormal structural change in dendritic spines is a pathological hallmark of neurodevelopmental and neuropsychiatric disorders, including depression (Pignataro et al., 2019). Thus, we investigated effects of c-MSST on spine morphology and plasticity in the V1 region of CUMS mice. Morphometric analysis (Figures 6A and 6B) of Golgi-Cox stained neurons showed that CUMS mice had lower spine density compared to control mice (Figures 6C and 6D). Notably, these morphological alterations were reversed by c-MSST (Figures 6C and 6D). Knockdown of ABCA1 in the V1 blocked the ability of c-MSST to reverse total dendritic spine density (Figures 6C and 6D). Specifically, our results confirmed that c-MSST protected dendritic morphology from chronic stress by elevating the densities of long thin, thin and mushroom dendritic spines, but not filopodia, stubby or branched dendritic spines (Figure 6E). CUMS procedure also induced a significant increase in the percentage of stubby dendritic spines and a significant decrease in the percentage of mushroom dendritic spines, and these effects were reversed by c-MSST (Figure 6F). Likewise, the beneficial effects of c-MSST on specific dendritic spines and proportions were blocked by neuron-specific knockdown of ABCA1 in CUMS mice (Figures 6E and 6F). Thus, maintenance of dendritic spine morphology and restoration of synaptic plasticity in the mouse V1 is dependent on the integrity of neuron ABCA1, which may underlie the antidepressant effects of c-MSST.

**Figure 7:**
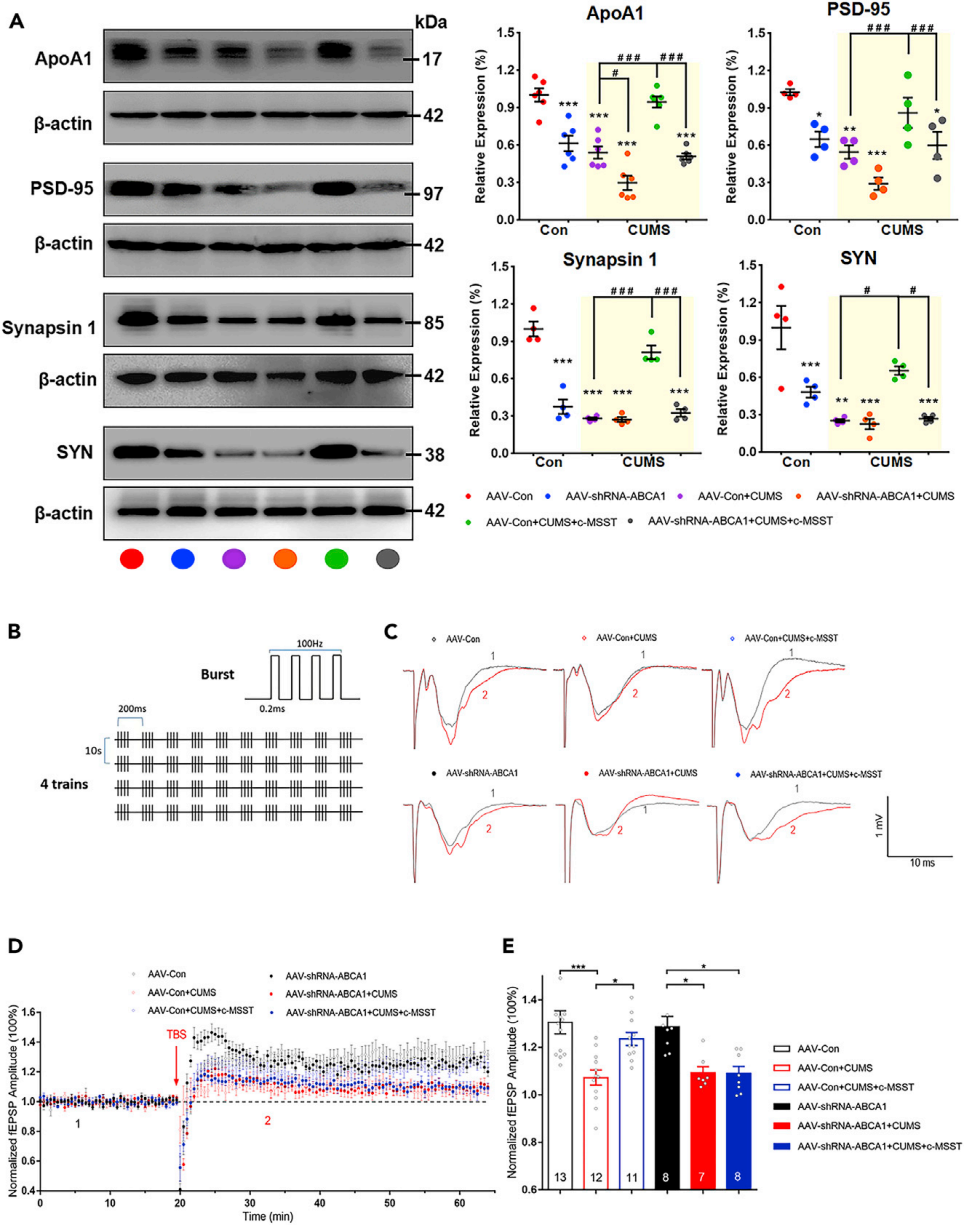
Neuron-specific knockdown of ABCA1 blocks the antidepressant effects of c-MSST via regulation of synaptic plasticity in the mouse V1 region (A) Representative immunoblots and bar graphs showing the expression of ApoA1, PSD-95, synapsin-1 and SYN in the mouse V1 after 5 days of 5 Hz c-MSST. n = four to six mice per group. Data are expressed as mean ±SEM. ∗p < 0.05, ∗∗p < 0.01, ∗∗∗p < 0.001 versus AAV-Con group, ^#^p < 0.05, ^###^p < 0.001 using ANOVA with Bonferroni correction. (B) TBS stimulation parameter. (C) Representative traces were taken at time points 1 and 2 indicated in the summary plots; fEPSPs recorded for each condition before (1) and 45 min after the application of TBS (2). (D and E) Averaged fEPSP amplitude and summary plots of LTP induced by TBS. n = 7–13 mice per group. Data are expressed as mean ± SEM ∗p < 0.05, ∗∗∗p < 0.001 using ANOVA with Bonferroni correction. c-MSST, combined magnetic stimulation system treatment; CUMS, chronic unpredictable mild stress; ApoA1, apolipoprotein A1; PSD-95, postsynaptic density-95; SYN, synaptophysin; fEPSP, field excitatory postsynaptic potentiates; TBS, theta-burst stimulation; LTP, long-term potentiation. See also Figures S6 and S7.

In addition, we observed that CUMS exposure downregulated the levels of synapse-associated proteins, including postsynaptic density 95 (PSD-95), synapsin one and synaptophysin (SYN) in the V1. These changes were significantly rescued by c-MSST (Figure 7A). Importantly, knocking down ABCA1 blocked the above-mentioned effects of c-MSST on the expression of synaptic proteins in CUMS mice (Figure 7A). Similar to the findings on synapse-associated proteins, the diminished protein expression of ApoA1 in CUMS mice was restored by c-MSST and this rescue was eliminated by ABCA1 shRNA AAV microinjection (Figure 7A). These observations led to speculation that c-MSST might improve CUMS-induced synaptic plasticity impairment in the V1 in a manner dependent on activation of ABCA1/ApoA1 signaling. In addition, to determine whether c-MSST or gene knockdown in V1 affects visual function in mice, we examined the visual evoked potential. However, the visual electrophysiological results showed that neither c-MSST nor gene knockdown in V1 impacted visual function in mice (Figures S6 and S7), indicating that these approaches may be safe and well tolerated independently of changes in visual function.

To test whether c-MSST regulates synaptic plasticity in the left V1, we examined LTP induced by theta-burst stimulation (TBS) (Figure 7B). Field excitatory postsynaptic potential (fEPSP) recording results showed that CUMS exposure suppressed LTP in the V1, and this effect was normalized by c-MSST (Figures 7C and 7D). The fEPSP amplitude in CUMS mice that received c-MSST was significantly augmented compared to that in CUMS mice, and this effect was blocked by ABCA1 knockdown in the V1 (Figures 7D and 7E). These physiological investigations suggest that CUMS induces V1 LTP deficit and that ABCA1 is necessary for c-MSST-induced functional restoration of LTP.

**Figure 8:**
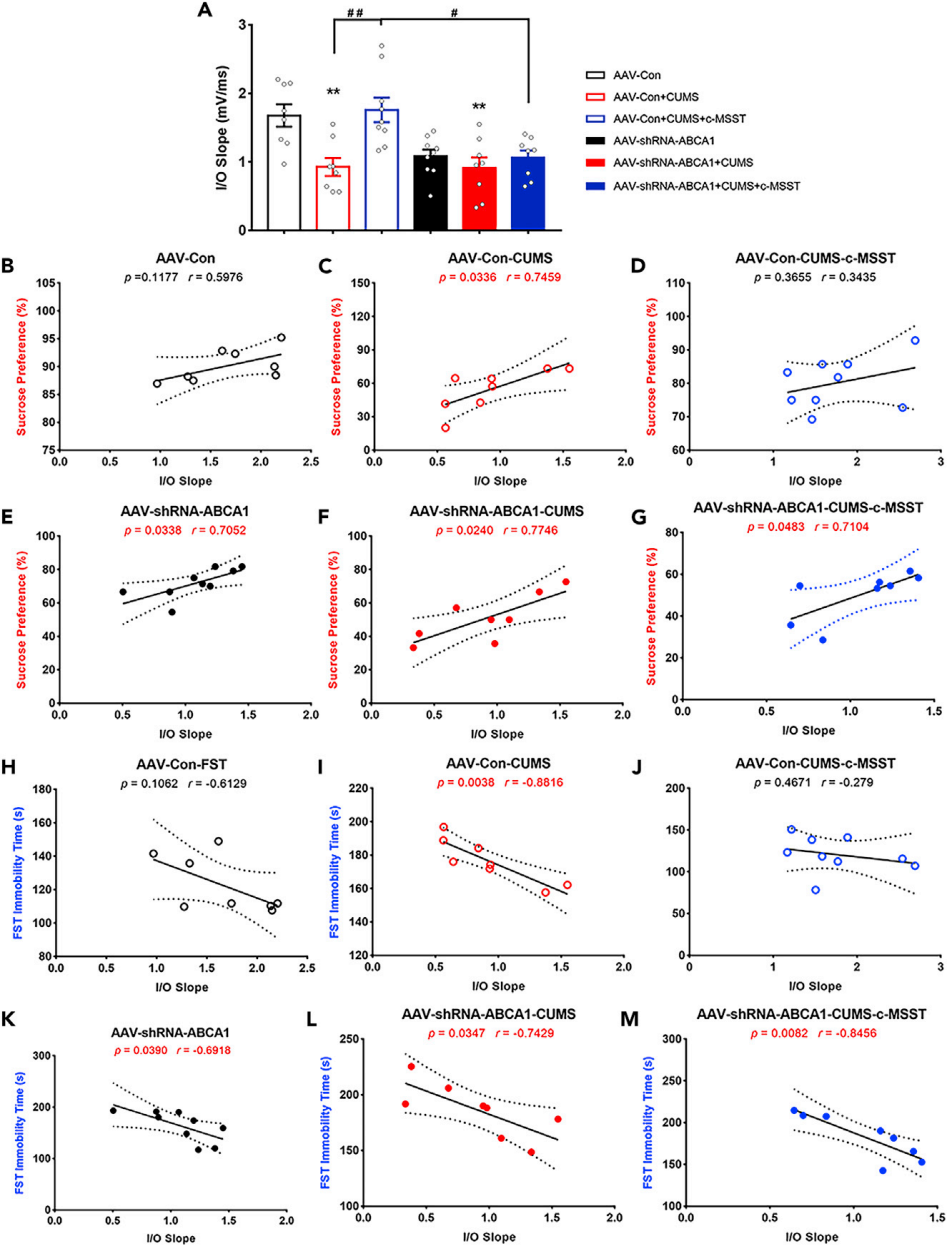
Correlation between the slope of input/output (I/O) curves and depression-like behaviors in mice (A) Bar graph depicting the maximal slope of I/O curves in different groups. n = eight to nine mice per group. Data are expressed as mean ±SEM. ∗∗p < 0.01 versus AAV-Con group, ^#^p < 0.05, ^##^p < 0.01 using ANOVA with Bonferroni correction. (B–M) AAV-Con group, black open circle; AAV-shRNA-ABCA1 group, black solid circle; AAV-Con + CUMS group, red open circle; AAV-shRNA-ABCA1 + CUMS group, red solid circle; AAV-Con + CUMS + c-MSST group, blue open circle; AAV-shRNA-ABCA1 + CUMS + c-MSST group, blue solid circle. Correlation between the slope of I/O curves and depression-like behaviors (SPT and FST) in different groups of mice analyzed by linear regression analysis and Pearson’s correlation (two-tailed). n = eight to nine mice per group. I/O, input/output; CUMS, chronic unpredictable mild stress; c-MSST, combined magnetic stimulation system treatment; SPT, sucrose preference test; FST, forced swim test. See also Figure S8.

Generally, input/output (I/O) curve is used as an approach to assess basal-synaptic transmission (Bayat et al., 2020; Woolley et al., 1997). Thus, we further examined the effects of c-MSST on I/O curves in the V1 region. In keeping with the results of fEPSP amplitude, there was a sharp drop in the I/O curve in CUMS mice compared to that in control group (Figure 8A). Importantly, c-MSST targeting the V1 recovered the I/O curve to levels comparable with control group, an effect that was completely eliminated by ABCA1 deficiency in the V1 (Figure 8A). Furthermore, correlation analysis showed significant positive correlations between I/O curve and sucrose preference in CUMS mice, ABCA1-knockdown mice that underwent CUMS, but not in unstressed mice or c-MSST CUMS mice (Figures 8B-8G). Conversely, we observed significant negative correlations between I/O curve and FST immobility time in depression-prone mice compared to control and c-MSST mice (Figures 8H–8M). However, no correlations were observed between I/O curve and any of other three behavioral parameters in all groups of mice (Figure S8).

## Discussion

The present study demonstrates that 5 days of 5 Hz magnetic stimulation of the V1 using a c-MSST improves depression-like behaviors in CUMS and LPS mouse models of depression. The molecular, morphological and functional investigations revealed that neuronal ABCA1/ApoA1 in the V1 is essential for c-MSST to preserve dendritic structure, dendrite spine density, synapse-associated proteins and LTP in the V1. Overall, these findings highlight the role of magnetic stimulation of visual cortex in rapid treatment of depression.

The specific strengths of this study are as follows: (1) We created the c-MSST to achieve magnetic stimulation of any targeted brain area in mice and found that 10Hz c-MSST targeting the left Prl cortex rapidly improved depression-like behaviors (Lu et al., 2020). The present study shows that c-MSST-induced magnetic stimulation of V1 induces consistent and observable antidepressant effects; (2) The disadvantages of rTMS treatment could be overcome by precise injection of SPIO nanoparticles under an external MF, thus enabling both deeper penetration and better focus on targeted brain area. Importantly, c-MSST-induced magnetic stimulation was precisely localized to the injected area of nanoparticles to play this role; (3) The underlying mechanism of c-MSST was analyzed and confirmed in four strictly matched groups. We showed that ABCA1/ApoA1 metabolic signaling pathway is required for c-MSST to exert antidepressant effects; (4) By overcoming the technical limitations of rTMS described above, c-MSST can achieve precise regulation of synaptic plasticity via accurate stimulation of specific brain regions in small animals. Overall, the technological innovations and multi-level validations performed in this study synergistically support reliability of the observed benefits of c-MSST.

Despite mounting evidence of abnormal visual cortex function in patients with depression, there are very few reports of direct intervention in the visual cortex to improve depression. Recently, our team found that individualized rTMS of the visual cortex results in therapeutic effects in MDD patients at day 5 (Song et al., 2020; Zhang et al., 2021). Similar to the results of our earlier clinical trial, direct stimulation of V1 with c-MSST required 5 days to induce antidepressant effects in the CUMS mouse model of depression, which is faster than light therapy and first-line antidepressant drugs. These observations support the potential of the visual cortex as an important brain region for observable treatment of depression. In depth clinical studies with independent large samples are needed to replicate these findings.

The pathogenesis of depression is not well understood and accurate molecular markers that can help diagnosis or predict efficacy of antidepressants are still lacking. Here we employed quantitative proteomics to explore the underlying mechanisms of c-MSST in ameliorating depression-like behaviors. Our bioinformatics and molecular biology analyses support that ApoA1 in the V1 significantly decreased after exposure to CUMS and was then rescued by c-MSST intervention. Recent studies have found that depleted levels of ApoA1 might may a role in the pathophysiology of various neurodegenerative disorders, such as Alzheimer’s disease (Lin et al., 2015) and depression (Morris et al., 2021). Electroconvulsive therapy is effective for the treatment of MDD and is accompanied by an increase in serum levels of ApoA1in MDD patients (Aksay et al., 2016). A large-scale meta-analysis found that lower levels of ApoA1 were associated with increased odds of depression (Bot et al., 2020). Thus, it is highly possible that the pathophysiology of depression may be closely associated with changes in circulating ApoA1 levels. In the present study, we found that ApoA1 was specifically co-localized with neurons and that neuronal levels of ApoA1 tended to be lower in the V1 region of CUMS mice. We speculate that decreased neuronal ApoA1 may be ascribed to lower circulating ApoA1 levels in depressive-like mice. In support of this hypothesis, our unpublished data reveal that plasma levels of ApoA1 are downregulated in both MDD patients and depressive-like mice and that plasma ApoA1 levels are negatively correlated with the severity of depressive symptoms and cognitive impairment in MDD patients. Importantly, the changes in plasma ApoA1 levels were able to predict the response to rTMS treatment in MDD patients, further supporting the potential of ApoA1 as a biomarker for depression.

Because cells in the CNS do not produce ApoA1, brain cells must acquire a significant amount of ApoA1 from the blood to maintain their functions via unknown mechanisms (Koch et al., 2001; Stukas et al., 2014). ABCA1 loaded the out flow of cholesterol and phospholipids from cells to lipid-poor apolipoproteins (ApoA1 and ApoE), thus generating nascent high-density lipoproteins (Dergunov et al., 2021). Moreover, disordered lipid metabolism because of dysregulated cholesterol efflux may be involved in depression-induced cognitive disorders (Ye et al., 2020). Similar to the results for ApoA1, the protein level of ABCA1 was downregulated in the V1 of CUMS mice and reversed by c-MSST stimulation. Our results further revealed an obvious decrease in cholesterol synthesis in the V1 region of CUMS mice that received c-MSST. Collectively, these results suggest that ABCA1/ApoA1 signaling pathways in neurons of the mouse visual cortex V1 region are selectively activated by c-MSST to reduce cholesterol overproduction, thereby rendering resistance to depressive-like behavior in mice.

An increasing body of evidence supports that synaptic plasticity in the human visual cortex plays a role in mediating the therapeutic actions of transcranial magnetic stimulation and antidepressants (Frase et al., 2021; Normann et al., 2007). Here, we focused on the effects of c-MSST on synaptic plasticity-related events in an animal model of depression. We found that synaptic plasticity was disrupted in the V1 of CUMS mice. Dysfunctional synaptic plasticity was ameliorated by c-MSST. Most importantly, we found that neuron-specific knockdown of ABCA1 abolished the therapeutic effects of c-MSST on depression-like behaviors and synaptic plasticity impairment. Collectively, these results imply that ABCA1/ApoA1 signaling pathway in the V1 is selectively activated by c-MSST, thereby rendering resistance to depression-like behaviors and synaptic dysfunction in mice. Moreover, we found correlations between I/O curves and depression-like behaviors in SPT and FST in CUMS mice, suggesting that impaired basal-synaptic transmission is closely associated with these depressive behaviors. Intriguingly, these correlations disappeared in CUMS mice treated with c-MSST in the V1, providing evidence linking dysfunctional basal-synaptic transmission to increased depression-like behaviors in mice. It should be noted that only *ex vivo* brain slices, not *in vivo* experiments, were obtained to ascertain the ameliorating effects of c-MSST on plasticity in the visual cortex because of the above-mentioned limitations of the technique.

In conclusion, our findings indicate that magnetic stimulation of the V1 may be a therapeutic approach for depression. This therapeutic strategy could expand the potential applications of physical MF therapy and promote brain research with clinical applications. Furthermore, our c-MSST offers potential for elucidation of brain activity mechanisms induced by rTMS in clinical settings. Considering the availability of visual cortex magnetic therapy, additional large-scale, randomized controlled clinical studies should be conducted to confirm the antidepressant effects of magnetic therapy in the visual cortex.

### Limitations of the study

This study demonstrated that c-MSST targeting the visual cortex induced rapid antidepressant effects, but there were several limitations in the present study. Although c-MSST realized precise stimulation of the visual cortex in small animals, it is still traumatic. We are trying to develop non-invasive stimulation of the visual cortex to achieve clinical treatment of depressive patients in the future. We also did not determine the underlying mechanism by whereas c-MSST ameliorated the depression-like behaviors in LPS-induced mice as it will be the focus of the future work. In addition, we did not investigate how this observation integrated with other known mechanisms of rTMS in neuroprotection, such as anti-inflammatory effects, anti-oxidative stress actions, enhancement of neurogenesis and inhibition of the hypothalamic-pituitary-adrenocortical axis.

## STAR★Methods

### Key resources table

**Table:** 

REAGENT or RESOURCE	SOURCE	IDENTIFIER
**Antibodies**		

Rabbit polyclonal anti-ABCA1	Abcam	Cat#ab7360; RRID:AB_305880
Mouse monoclonal anti-PSD95	Abcam	Cat#ab13552; RRID:AB_300453
Rabbit polyclonal anti-Synapsin	Abcam	Cat#ab64581; RRID:AB_1281135
Rabbit monoclonal anti-SYN	Abcam	Cat#ab32127; RRID:AB_2286949
Mouse monoclonal anti-actin-β	Abcam	Cat#ab8226; RRID:AB_306371
Rabbit monoclonal anti-NeuN	Abcam	Cat#ab236870
Goat anti-Mouse IgG (H+L) Highly Cross-Adsorbed Secondary Antibody, Alexa Fluor 594	Thermo Fisher Scientific	Cat# A-11032, RRID:AB_2534091
Goat anti-Rabbit IgG (H+L) Highly Cross-Adsorbed Secondary Antibody, Alexa Fluor Plus 594	Thermo Fisher Scientific	Cat# A32740, RRID:AB_2762824
Goat anti-Rabbit IgG (H+L) Cross-Adsorbed Secondary Antibody, Alexa Fluor 488	Thermo Fisher Scientific	Cat# A-11008, RRID:AB_143165
Anti-mouse IgG, HRP-linked Antibody	Cell Signaling Technology	Cat#7076; RRID:AB_330924
Anti-rabbit IgG, HRP-linked Antibody	Cell Signaling Technology	Cat#7074, RRID:AB_2099233
Rabbit polyclonal anti-iba-1	FUJIFILM Wako Shibayagi	Cat#019-19741; RRID:AB_839504
Rabbit polyclonal anti-GFAP	Abcam	Cat#ab7260; RRID:AB_305808
Mouse monoclonal anti-ApoA1	Thermo Fisher Scientific	Cat#MIA1404; RRID:AB_11151929
Mouse monoclonal anti-ABCA1	Abcam	Cat#ab66217; RRID:AB_1141083

**Bacterial and virus strains**		

rAAV2/9-hsyn-EGFP-5'miR-30a-shRNA(scramble)-3′-miR30a-wpres	BrainVTA, Wuhan	This paper
rAAV2/9-hsyn-EGFP-5’miR-30a-shRNA(Abca1)-3′-miR30a-wpres	BrainVTA, Wuhan	This paper

**Biological samples**		

Human blood	Second Affiliated Hospital at Xinxiang Medical University and Affiliated Zhongda Hospital of Southeast University	NA

**Chemicals, peptides, and recombinant proteins**		

Lipopolysaccharides from *Escherichia coli* O 127:B8	Sigma	Cat#L4516
Mounting Medium With DAPI	Abcam	Cat#ab104139

**Critical commercial assays**		

TaKaRa MiniBEST UniversalRNA Extraction Kit	TaKaRa	Cat# 9767
FD Rapid Golgi Stain Kit	FD NeuroTechnologies	Cat# PK401
Click-iT™ Plus TUNEL Assay	Thermo Fisher Scientific	Cat# C10617
HiScript II OneStep qPCR-PCR-SYBR Green Kit	Vazyme	Cat# Q221-01
Human ApoA1(Apolipoprotein A1) ELISA Kit	Elabscience	Cat#E-EL-H0125c
Mouse ApoA1(Apolipoprotein A1) ELISA Kit	Elabscience	Cat#E-EL-M3016
Total cholesterol assay kit	Nanjing Jiancheng Bioengineering Institute	Cat#A111-1-1

**Deposited data**		

Raw and analyzed data	This paper	ProteomeXchange Consortium, PRIDE: PXD031598

**Experimental models: organisms/strains**		

Mouse: C57BL6/J	Sino-British SIPPR/BK Lab Animal	N/A

**Oligonucleotides**		

shRNA2(Abca1)GGGTGTCAGTAATTCTCAAGC	BrainVTA, Wuhan	PT-2975
shRNA3(Abca1)GCGTGAAGCCTGTCATCTACT	BrainVTA, Wuhan	PT-2976
shRNA (scramble)CCTAAGGTTAAGTCGCCCTCG	BrainVTA, Wuhan	PT-0961
Real-time PCR primers	Tsingke Biotechnology	This paper
shRNA1(Abca1) GGAGTTGGCTGTGTTCCATGA	BrainVTA, Wuhan	PT-2974

**Software and algorithms**		

Any-maze	Stoeling	RRID:SCR_014289
GraphPad Prism version 7.0	GraphPad Software	RRID:SCR_002798
ImageJ	NIH	https://imagej.net/
Photoshop CC	Adobe	RRID:SCR_014199
Microsoft Excel	Microsoft	RRID:SCR_016137
SPSS	IBM	RRID:SCR_002865

**Other**		

Magnetic field generator	Southeast University	This paper
Superparamagnetic iron oxide (SPIO) nanoparticles	Zhengda Tianqing Pharmaceutical Co., Ltd	NA

### Resource availability

#### Lead contact

Further information and requests for resources and reagents should be directed to and will be fulfilled by the Lead Contact, Zhijun Zhang (janemengzhang@vip.163.com).

#### Materials availability

This study did not generate new unique reagents.

### Experimental model and subject details

#### Animals

Male adult C57BL/6 JAX™ mice purchased from Sino-British SIPPR/BK Lab Animal Ltd. (Shanghai, China) were used in this study. Mice were housed under a constant ambient temperature (23 ± 1°C) and relative humidity (55 ± 2%). Four mice were housed per cage under a 12 h light/dark cycle (light on at 07:00 AM) and provided food and water *ad libitum.* Mice were allowed to habituate to our laboratory facilities for 2 weeks prior to experiments. Body weight and coat scores were measured every 5 days. The sum coat scores included seven different body parts: head, neck, dorsal coat, ventral coat, forepaws, hind paws and tail. One point was awarded for an unkempt coat in each area; thus, a higher coat score indicates more severe depressive symptoms. All animal care and experimental procedures were performed in strict accordance with the appropriate institutional ethical guidelines and approved by the Institutional Animal Care and Use Committee of Southeast University (approval number: 20180501006).

#### Human experiments

Clinical information and blood sample of MDD patients and healthy control subjects were collected from the Second Affiliated Hospital at Xinxiang Medical University and Affiliated Zhongda Hospital of Southeast University. The research protocol was approved by the local ethics committee of local ethics research board (Ethical Approval Number: 2016ZDSYLLl00-P01) and all participants (or parents or legally authorized representatives) provided written informed consent. The trial design and detailed methods are published by our research group (Zhang et al., 2021). Detailed demographic information and behavior measurements by all subjects at baseline were listed in Table S1. Subjects enrolled in this study were randomly allocated in two groups and underwent 5-day (twice per day) real and sham visual cortical rTMS, without any other therapies during the trial. MRI was performed on a 3.0 T MRI scanner (Siemens, Verio) to ensure accurate positioning of the rTMS coil on the stimulation site. Sham stimulation was delivered putting the coil upside-down. The relevant rTMS parameters were defined as follows: stimulation intensity, 90% resting motor threshold (RMT); frequency, 10 Hz; train duration, 4 sec; inter-train interval, 26 sec; pulse number, 1600/session, total duration, 20 min. Behavior measurements after 5-day treatment were described in the Table S2.

### Method details

#### Establishment of c-MSS

The c-MSS consisted of SPIO nanoparticles and a magnetic field (MF). The γ-Fe_2_O_3_ nanoparticles (tradename: Ferumoxytol) were manufactured by Jiangsu Zhengda Tianqing Pharmaceutical Co., Ltd. and approved by the Chinese Food and Drug Administration (CFDA) for clinical applications. Mice were anaesthetised with 2–3% isoflurane and secured with an ear bar in a mouse brain stereotaxic apparatus (RWD Life Science) for microinjection. A Hamilton syringe (10 μL, Hamilton Company) with a 33-gauge blunt-end needle was inserted into the brain for SPIO nanoparticle injection. The coordinates of the V1 were as follows: 0.1–0.3 mm anteroposterior (A/P); ±1.7–2.2 mm mediolateral (M/L); and −0.7 mm dorsoventral (D/V) from lambda (Hammad et al., 2015). The injection volume of SPIO nanoparticles was 300 nL with a 50 nL/min speed in the left V1. The needle was kept in site for 10 min and slowly withdrawn after completion of the injection. Next, the mouse was fixed in the prone position with its heads in an aluminium holder under the pulsed MF. Under the combined effect of the MF and SPIO nanoparticles, the left V1 was stimulated and activated while the animal was awake.

#### c-MSST and mouse models of depression

Briefly, c-MSST consists of SPIO nanoparticles (300 nL, 1.67 mg/mL) being stereotaxically injected into the left V1of a mouse that is fixed in the prone position with its head in an aluminium holder under a pulsed MF. Under the combined effect of the MF and SPIO nanoparticles, the left V1 is stimulated and activated while the mouse is in an awake state. The trace and safety results of the nanoparticles were assessed after microinjection into the left V1 via MRI and TUNEL staining. .

#### Observation of SPIO nanoparticles by MRI

MRI was performed to trace the location of the SPIO nanoparticles in the left V1 in mice on days 1, 3, 5 and 7 after microinjection. All MRI studies were conducted with a Bruker 7 Tesla system (Bruker Biospin, Ettlingen, Germany). A 72 mm diameter volume coil and a decoupled quadrature surface mouse brain coil (Bruker, Germany) were used for signal transmission and reception. Mice were anaesthetised and maintained by inhalation of isoflurane, N_2_O and O_2_ mixed gas and then placed in the MRI scanner and fixed with a head frame. T2-weighted (T2W) MR images were obtained in three planes (transverse, coronal scout, and sagittal) with the following parameters: 2 cm × 2.3 cm field of view (FOV); 0.256 mm × 0.256 mm matrix (MTX); 0.7 mm slice thickness (SI); 18 contiguous coronal slices; 42.4 ms echo time (TE); and 3000 ms repetition time (TR).

#### Safety assessment of SPIO nanoparticles

To assess the safety of SPIO nanoparticles and c-MSST in mice V1 brain tissue, the Click-iT Plus TUNEL assay kit (Invitrogen Thermo-Fisher, Shanghai, China) was used. According to the manufacturer’s protocol, the sectioned visual cortex tissue on a slide was fixed with 4% formaldehyde solution for 15min at 37°C followed by incubation with proteinase K solution for 15 min. After rinsing the slides in deionised water, the slides were immersed in the TUNEL reaction mixture at 37°C for 60 min and counterstained with 4′,6-diamidino-2-phenylindole (DAPI) (Abcam) in the dark. Lastly, the slides were visualised under a confocal fluorescence microscope.

#### CUMS- and LPS- induced depression-like mice

The chronic stress group mice were individually housed and underwent a variable sequence of unpredictable and mild stimuli for 5 weeks (total of 35 days). The stimuli included warm water swimming (45°C for 5 min), cold water swimming (4°C for 5 min), restraint stress for 2 h, cage shaking for 5 min, tail pinching for 1 min, tail suspension for 6 min, food deprivation for 12 h, water deprivation for 12 h, moist bedding for 12 h, overnight illumination for 12 h, cage tilt (45°) for 10 h, empty cage for 12 h, stroboscopic illumination for 12 h and rat bedding for 24 h. The CUMS mice experience two types of mild stimuli each day that were unpredictable in time and manner. Mice in the control condition were handled except for the necessary procedures, such as routine cleaning and behavioural tests. Only depression-susceptible mice that displayed a ±10% change from baseline in all behavioural tests were used for the subsequent experiments. The LPS-induced depression model in mice has been broadly validated. LPS (serotype 0127:B8) was purchased from Sigma-Aldrich. LPS (1 mg/kg) or saline was injected intraperitoneally (i.p.) for 5 successive days. Behavioural tests were performed 24 h after the last administration of LPS.

#### Sucrose preference test (SPT)

The SPT was used to assess anhedonia, which is a core symptom of depression in animals. To adjust to drinking the sucrose solution, each group was accustomed to drinking from two bottles of 1% (w/v) sucrose solution for 48 h. Next, the sucrose bottle was replaced with a bottle of water for 48 h before the CUMS or LPS procedure. Mice were subjected to SPT 1 day before the CUMS procedure and at the end of the CUMS or LPS procedure. One mouse per group was placed in the test cage and fasted (no food or water) for 24 h, and then given one bottle filled with 1% (w/v) sucrose solution and one bottle filled with water for 2 h. The position of the two bottles was changed after 1 h. At the end of the SPT, the weight of the liquid consumed was measured. The sucrose preference value was normalised as a ration of sucrose solution intake to the combination of sucrose solution intake with water intake.

#### Forced swim test (FST)

The FST is a well-established despair-based test used to examine depressive behaviour. Briefly, mice were individually forced to swim in a transparent glass cylinder (16 cm in diameter, 25 cm in depth) filled with fresh water (23–25°C) to a depth of 20 cm for 6 min. The immobility time during the last 4 min was measured. Immobility was characterised as follows: no movement except for the necessary movement made by the mouse to keep its head above the water. A longer immobility time indicated a more severe depression-like state. All experimental mice were wrapped in dry towels and dried with a hairdryer immediately after the FST.

#### Tail suspension test (TST)

The TST is another widely used despair-based test. For this test, each mouse was individually suspended 50 cm above the floor using adhesive tape placed approximately 2 cm from its tail tip for 6 min. The immobility time during the last 4 min was assessed. A longer immobility time indicated a more severe depression-like state.

#### Open field test (OFT)

Locomotor activity, exploratory behaviour and anxiety were assessed using the OFT. A custom-made square box (50 × 50 × 50 cm) was placed in a darkened and sound-proofed room with the bottom of box divided into 16 equal small areas. Mice were individually placed in the centre of the apparatus for a 5min period. The distance travelled, speed and time spent in each of the subdivisions were used as the test parameters. Between tests, the OFT apparatus was cleaned with 75% ethanol solution to remove any olfactory cues. The FST, TST and OFT data were calculated and analysed using the ANY-MAZE behavioural monitoring system (Stoelting Co., Wood Dale, IL, USA). All behavioural tests were carried out by an experimenter blinded to the treatment status.

#### Proteomic analysis

##### Protein extraction and digestion

Frozen left V1 tissues were ground into powder in liquid nitrogen and lysed in SDT buffer (4% (w/v) SDS, 100 nM Tris/HCl, pH 7.6, 0.1 M DTT). Next, the samples were sonicated on ice and the supernatants were collected following centrifugation (10,000 g, 30 min, 4°C). Protein concentrations were quantified using the enhanced bicinchoninic acid (BCA) protein estimation kit (P0010; Beyotime, Jiangsu, China). Proteins were digested based on the filter aided sample preparation (FASP) method. The digested peptides were desalted using a C18 solid phase extraction cartridge. Lastly, the samples were lyophilised and redissolved in dissolution buffer.

##### iTRAQ labelling and SCX separation

A total of 100 μg peptide samples were individually labelled using the 8-plex iTRAQ multiplex kit following the manufacturer’s protocol (AB Sciex, USA). Reagents 114, 115, 116 and 117 were used to label the control, CUMS, CUMS + SPIO and CUMS + c-MSST groups, respectively. To increase peptide detection, the mixed iTRAQ-labelled peptides were fractionated using strong cation exchange (SCX) chromatography. SCX chromatography was performed. The peptide mixture was bound with buffer A (pH 3.0) (10mM KH_2_PO_4_ in 25%ACN) and eluted with buffer B (pH 3.0) elution (10mM KH_2_PO_4_ and 500mM KCl in 25% ACN). Each fraction was desalted using a C18 column (Millipore) and prepared for liquid chromatography-tandem mass spectrometry (LC-MS/MS) analysis.

##### LC-MS/MS analysis

The fractions were separated using an EASY-nLC 1000 system (Thermo Fisher Scientific). The binary buffer system included an A phase (0.1% formic acid in 2% acetonitrile) and B phase (0.1% formic acid in 84% acetonitrile). Peptides were re-dissolved in the A phase and loaded into a reversed-phase analytical pre-column (Acclaim PepMap100, 100 μm inner diameter × 2 cm, Nanoviper® C18 separation column, Thermo Fisher Scientific). Next, each fraction was directly injected into and analysed by the Easy Column (10 cm × 79 μm, 3 μm particle size, Thermo Fisher Scientific) at a flow rate of 330 nL/min. The LC-MS/MS analysis was performed using a Thermo Fisher Scientific Q-Exactive high resolution mass spectrometer. The MS scan was set in the positive ion mode (300–1800 *m*/*z*). The resolutions of MS1 and MS2 were 70,000 at 200 *m*/*z* and 17,500 at 200 *m*/*z*, respectively. For MS1, the automatic gain control (AGC) target value, maximum ion injection time and dynamic exclusion were set to 1 × 10^6^, 50 ms and 60 s, respectively. MS data were acquired by full-MS scan followed by 20 MS2 spectra. For MS2, each activation type was HCD and the collision energy was 30 eV for all ions. The isolation window and underfill ratio were defined as 2*m*/*z* and 0.1%, respectively.

##### Protein identification and quantification

All LC-MS/MS data were analysed using the MASCOT database (Mascot Daemon Version 2.2, Matrix Science, London, UK) and Proteome Discoverer Software (Version 1.4, Thermo Fisher Scientific). For protein identification, the following search parameters were set: cleavage enzyme = trypsin; max missed cleavages = 2; fixed and variable modifications = carbamidomethyl (C) and oxidation (M); peptide mass tolerance = ±20 ppm; fragment mass tolerance = 0.1 Da; and peptide false discovery rate (FDR)≤ 0 01. Only the unique and non-confrontational peptides in a protein were utilised to calculate the protein abundances and ratios. The median protein ratio was used to normalise all peptide ratios. The differential expressed proteins (DEPs) (up-regulated and down-regulated) were determined using a 1.2-fold (> 1.20 or < 0.83) change cutoff with a p-value < 0.05 (with a 95% confidence interval).

##### Bioinformatics analysis

The differential expressed proteins (DEPs) were analysed by hierarchical cluster analysis (HCA) using Cluster 3.0 software and visualised in the form of heat map. Gene ontology (GO) analysis (http://www.gneontology.org) and KEGG analysis (http://www.genome.jp/kegg/pathway.html) were performed to categorise the biological functions (cellular component, biological processes and molecular functions) of the DEPs and pathways they were involved in. Protein-protein interaction (PPI) network analysis was performed using the Search Tool for the Retrieval of Interacting Proteins (STRING) software (https://www.stringdb.org/).

#### Protocols of recombinant AAV

After transfection to B16 cells for 72 h (2 μg per well), the interference efficiency was confirmed by RT-PCR. The PCR primer sequences were as follows: ABCA1: 5′-AAGCCGCGAAGGAGGGAGCC-3′; reverse, 5′-GGCCCGTCTGTTTCGTCTTGCTG-3′, GAPDH: forward, 5′-GCCCATCACCATCTTCCAGGAGCG-3′; and reverse, 5′-GCAGAAGGGGCGGAGATGATGACC-3′. Data were quantified using the ΔΔCt method and normalised to ABCA1/GAPDH expression. The sequence for ABCA1 shRNA with the highest interference efficiency was 5′-GCGTGAAGCCTGTCATCTACT-3′, and the sequence for the scramble shRNA was 5′-CCTAAGGTTAAGTCGCCCTCG-3′. The final recombinant AAV-ABCA1 knockdown cassette was rAAV-hsyn-EGFP-5’miR-30a-shRNA(Abca1)-3′-miR30a-wpres. All viral particles were packaged under the control of the synapsin-1 (SYN) promoter (a neuron-specific promoter). The titerres of all viruses were verified by quantitative polymerase chain reaction (qPCR). It was estimated that the stocks of AAV-shRNA-ABCA1 have a titre of 2.05 × 10^12^/mL virus particles. All aliquots were stored at −80°C until use.

#### Immunoblot analysis

Mice were sacrificed under deep anaesthesia with 1% pentobarbital immediately after the final behavioural test. The entire tissue of the left V1 was lysed in RIPA lysis buffer and collected after centrifugation at 12,000 rpm for 15 min. Quantification of protein in supernatants was performed using the BCA assay (Thermo Fisher Scientific). Equal amounts of protein extracts were separated by SDS-PAGE and transferred to polyvinylidene difluoride (PVDF) membranes (Millipore, Bedford, MA). Membranes were blocked and probed with following appropriate antibodies according to standard procedures: ABCA1 (1:1,000, Abcam, ab7360), ApoA1 (1:1,000, Invitrogen, MIA1404), PSD-95 (1:1,000, Abcam, ab13552), Synapsin I (1:1,000, Abcam, ab64581), Synaptophysin (1:20,000, Abcam, ab32127) and β-Actin (1:1,000, Abcam, ab8226). After incubation with horseradish peroxidase (HRP)-conjugated secondary antibody for 1 h at room temperature, the bands were visualised using enhanced chemiluminescence (ECL) according to the manufacturer’s protocols. Protein quantification was obtained using ImageJ software (http://imagej.net/ImageJ) and the specific protein expression level was normalised to the level of β-actin on the same PVDF membrane.

#### Immunofluorescence

For immunohistochemistry staining, mice were deeply anaesthesia with 1% pentobarbital after the final behavioural test and perfused with cold phosphate-buffered saline (PBS) for 5 min and then with 4% paraformaldehyde (PFA) in PBS for 5 min transcardially. The whole brain was removed and fixed overnight in 4% PFA in PBS, then dehydrated in gradient sucrose (20–30%) for 48h at 4°C. The brain was then embedded with Tissue-Tek OCT compound (Sakura) in liquid nitrogen and stored at −80°C. Next, 16 μm thick coronal sections from the brain samples were cut using a microtome (CM1900, Leica, Nussloch, Germany) and mounted on poly-lysine-treated glass slides. All staining procedures were performed in dark conditions. Brain slices were permeabilised in PBS with 0.2% Triton X-100 and 0.5% bovine serum albumin (BSA) for 10 min and blocked with 2.5% goat serum in PBS with 0.2% Triton X-100 and 0.5% BSA for 2 h. This was followed by incubation overnight at 4°C with primary mouse ApoA1 antibodies (1:500; Invitrogen, MIA1404), mouse ABCA1 antibodies (1:500, Abcam, ab66217), rabbit NeuN antibodies (1:100, Abcam, ab236870), rabbit iba-1 antibodies (1:500, Wako, 019–19741) and rabbit GFAP antibodies (1:500, Abcam, ab7260). After five washes in PBS, brain slices were incubated for 2 h with fluorochrome-conjugated secondary antibodies (Thermo Fisher Scientific). After another five washes in PBS, the slides were sealed with DAPI containing mounting medium (AB104139, Abcam). Co-localization confocal fluorescence images were obtained and analyzed using an Olympus FV3000 microscope (Olympus, Tokyo, Japan).

#### Golgi-Cox staining and analysis

The mouse brains were first immersed in Golgi solution (A + B, A/B = 1:1, 15 mL/mouse) for 14 days and then incubated in Golgi solution C for 3 days at room temperature in dark conditions. Next, the brains were coronally sectioned at 100 μm slices with a Leica VT100S vibrating microtome (Wetzlar, Germany) and mounted on gelatine-coated slides. After incubation in a solution containing silver nitrate and dehydration, slides were covered with Permount Mounting Medium and cover slips. Finally, images of dendritic spines were captured by microscopy with a 100 × objective (Olympus DP73, Tokyo, Japan). Spine density was counted in the secondary apical dendrites of pyramidal neurons from the V1 and measured using Image J software. About 40 pyramidal neurons chosen from 5 mice were quantified in a blinded manner for morphological analysis.

#### ELISA assay

Blood samples (1.5 mL) were placed in heparinized tubes and centrifuged to obtain plasma. ApoA1 in plasma from mice and clinical subjects was measured by mouse enzyme-linked immunosorbent assay (ELISA) kit (Elabscience, #E-EL-M03016) and human ApoA1 ELISA kit (Elabscience, #E-EL-H0125c) according to the manufacturer’s protocol.

#### Biochemical analysis

Total cholesterol (TCHO) from mouse left V1 were measured by using commercial assay kits (Nanjing Jiancheng Bioengineering Institute, Nanjing, China) according to the manufacturer’s instructions.

#### Filipin staining

Filipin (GMS80059.3v.A, GenMed Scientifics Inc, USA) staining was carried out to monitor the levels of free cholesterol from mouseV1 according to the manufacturer’s instructions. The sections of the brain tissue (10 μm) were stained with filipin fluorescence dye. Images were captured using a confocal Olympus FV3000 microscope (Olympus, Tokyo, Japan) and the fluorescence intensity from each section was measured using Image J software.

#### Electrophysiological recordings

##### Solutions

The following external solutions were used for the electrophysiological experiments: Artificial cerebrospinal fluid for slicing (slicing aCSF) (mM): 125 NaCl, 2.5 KCl, 1.25 NaH_2_PO_4_, 25 NaHCO_3_, 10 D-glucose, 2.0 CaCl_2_ and 1.5 MgCl_2_. The solution was saturated with carbogen (95% O_2_/5% CO_2_) and kept ice-cold prior to use. Artificial cerebrospinal fluid for incubation (incubation aCSF) (mM): same formula as slicing aCSF. Solution was saturated with carbogen (95% O_2_/5% CO_2_) and kept at 30°C prior to use. Artificial cerebrospinal fluid for recording (recording aCSF) (mM): 125 NaCl, 2.5 KCl, 1.25 NaH_2_PO_4_, 25 NaHCO_3_, 10 D-Glucose, 2.0 CaCl_2_ and 1.0 MgCl_2_. Solution was saturated with carbogen (95% O_2_/5% CO_2_) and kept at 30.5 ± 1°C prior to use. Pipette solution: same formula as recording aCSF at room temperature. Anaesthetics: 25% (w/v) urethane, diluted in ddH_2_O. Agar plate: 3% (w/v) agar prepared in ddH_2_O, boiled and melted.

##### Preparation of brain slices

Acute coronal brain slices (380 μm thickness) were prepared from each group. Mice were deeply anaesthetised by i.p. injection of urethane (25%, 1 mL/100g) and decapitated. The brain was rapidly extracted from the skull with the least amount of damage and pressure on the brain. Next, the brain was transferred into ice-cold slicing aCSF for rapid cooling. The sample was glued to a specimen plate and mounted on a microtome (Vibratome VT1200S, Leica, Germany). The temperature in the buffer tray was kept at 0–4°C by replacing the aCSF. The prepared slices were recovered in incubation aCSF at 30°C for 30 min and then incubated at room temperature for an additional 1.5h. Throughout the whole process, aCSF was saturated with carbogen (95% O_2_/5% CO_2_).

##### Electrophysiological recordings

Electrophysiological recordings were obtained under visual control using an upright microscope (BX50-WI, Olympus, Japan). The infrared phase contrast made it possible to visualise neurons within a brain slice. The microscope was equipped with 10× ocular and 5× and 40× objectives. The microscope was installed on an anti-vibration table (PM-H-10-08-L, Ruixuguangdian, China) and surrounded by a Faraday cage. It was possible to record and display the image, which was controlled by an infrared-sensitive CCD camera (Retiga ELECTRO, QImaging, Canada). The brain slices were transferred to a “submerged” type recording chamber. Throughout the experiment, the brain slices were kept on a nylon net in a fixed position and covered by short silver wire loads. Outside the Faraday cage, in a thermostat water bath (HH-501, Xinbao Equipment, China), the recording aCSF was continuously saturated with carbogen (95% O_2_/5% CO_2_). The recording chamber was continuously perfused with aCSF by a peristaltic pump (BT100-2J, LongerPump, China) at a rate of 7 mL/min at 30.5 ± 1°C. Recordings were acquired using an Axopatch 700B amplifier and Digidata 1440A (Molecular Devices, USA). Clamp10.7 software was used for the acquisition of the recordings. Recording pipettes were pulled from thin wall borosilicate glass with a filament (BF150-110-10, Sutter Instrument, USA). The pipettes were produced with electronically electrode extracting equipment (P97, Sutter Instrument, USA).

For the field excitatory postsynaptic potential (fEPSP) recordings, a stimulation pipette was placed at visual cortex layer II/III at a 75–150 μm depth into the tissue. The electrode resistance amounted to approximately 1–2 MΩ after filling with recording aCSF. The recording electrode (approximately 1–2 MΩ) was placed on visual cortex layer IV, approximate 20 μm away from the stimulation pipette. The stimulating electrode was controlled by a mechanical micromanipulator (CFT-8301B, Jiangsuruiqi, China), and the recording electrode was controlled by a motorised micromanipulator (MP225, Sutter Instrument, USA).

To induce the fEPSP response, square-wave current pulses (100 μs pulse width) were delivered via a stimulus isolator (Isoflex, AMPI, Israel) to the stimulation electrode. All signals were low-pass filtered at 2 kHz. The stimulation intensity was adjusted through the stimulus isolator to produce a response of approximately 0.4–0.5mV. The typical waveform of the cortical fEPSP should have a duration of approximately 5–10 ms with a smooth decay phase. The response was regarded to be stable if the amplitude fluctuated less than 10% in 3 min. The input-output relationship (I-O curves) for synaptic transmission was tested by stimulating layer IV–layer II/III with increasing levels of intensity in 0.01 mA steps until the response reached the maximum. The maximum was determined by gradually increasing the stimulation intensity in steps. If no increase in response amplitude was observed after 2 or 3 consecutive times, the response was thought to have reached the maximum.

For the LTP tests, pulses at an intensity eliciting approximately 40–60% of the maximal response were delivered as the baseline level. The slices were stimulated with single test pulses every 30 s. After recording a stable baseline for at least 20 min, LTP was induced by theta-burst stimulation (TBS). TBS consisted of four trains delivered at 0.1 Hz, each train consisting of 10 bursts at 5 Hz and each burst delivering four stimuli at 100 Hz with a 200 μs stimulus duration. Following TBS, the stimulus parameter was returned to baseline for another 45 min recording. Approximately 35–45 min after stimulation corresponded to early phase LTP. At the beginning of the experiments, the input/output (I/O) curves were generated (average of 3 pulses with a duration of 200μs from1 to 8 V in steps of 1 V at 0.033 Hz) to determine the voltages used in the LTP experiments.

##### Data analysis

The I/O slope was measured by linear regression of the I/O curve. The slope and the amplitude were measured by fitting a straight line of 25%–75% of the fibre volley to the fEPSP peak using Clamfit10.7 software. For the scatter plot, the mean value of the amplitude over the baseline period was calculated. Next, each fEPSP response was standardised to a ratio of amplitude of each fEPSP to the mean baseline amplitude. For the histogram, the mean value of the last 10 min of the baseline was calculated to demonstrate stability. The mean value of the last 10 min after TBS (approximately 35–45 min) was used to identify whether LTP was successfully induced or not. To compare long-term synaptic plasticity between groups, the differences were statistically compared between the last 10 minutes after TBS induction.

#### Visual evoked potentials

Flash visual evoked potential (F-VEP) signals were used to evaluate visual function. Mice were dark-adapted overnight (>12 h) and anesthetized using ketamine and xylazine mix (95 mg/kg ketamine and 10 mg/kg xylazine) via intraperitoneal (i.p.) injection. Mice were placed in the heated surface with a Ganzfeld dome and recorded signals with Celeris system (Diagnosys LLC, Lowell, MA). A reference electrode was placed in snout whereas the ground electrode was placed at the tail root, measuring subdermal platinum electrodes were inserted at the location of the visual cortex. Each Eye was stimulated using white light flashes of 0.5 cds/m^2^ intensity respectively. Mice F-VEP signals were recorded for 300 ms at 2000 Hz. Experiments were conducted blind to all treatment condition.

### Quantification and statistical analysis

All statistical analyses were performed using GraphPad Prism Software (version 7; GraphPad Software, Inc.). Results are reported as the mean ± standard error of the mean (SEM). Differences among multiple groups were compared using analysis of variance (ANOVA). Differences between two groups were further analysed using the Sidak or Bonferroni post-hoc test. Further statistical details are provided in the respective figure legends. p< 0.05 was considered to be statistically significant.

## Data Availability

• The mass spectrometry proteomics data have been deposited at the Proteome Xchange Consortium and are publicly available of the date of publication (http://proteomecentral.proteomexchange.org/cgi/GetDataset?ID=PXD031598). Accession numbers are listed in the Key resources table. All data reported in this paper will be shared by the lead contact upon request. • This paper does not report original code. • Any additional information required to reanalyze the data reported in this paper is available from the Lead contactupon request.
