# Supplementary material for: Selective activation of ABCA1/ApoA1 signaling in the V1 by magnetoelectric stimulation ameliorates depression via regulation of synaptic plasticity
Source: iScience. 2022 Apr 4;25(5):104201. doi: 10.1016/j.isci.2022.104201 (PMC9036135; doi:10.1016/j.isci.2022.104201)
Supplement: Document S1. Figures S1–S8 and Tables S1 and S2 [file mmc1.pdf]

iScience, Volume *25*

Supplemental information

Selective activation of ABCA1/ApoA1 signaling in the V1 by magnetoelectric stimulation ameliorates depression via regulation of synaptic plasticity

Qingbo Lu, Fangfang Wu, Jiao Jiao, Le Xue, Ruize Song, Yachen Shi, Yan Kong, Jianfei Sun, Ning Gu, Ming-Hu Han, and Zhijun Zhang

SUPPLEMENTARY DATA

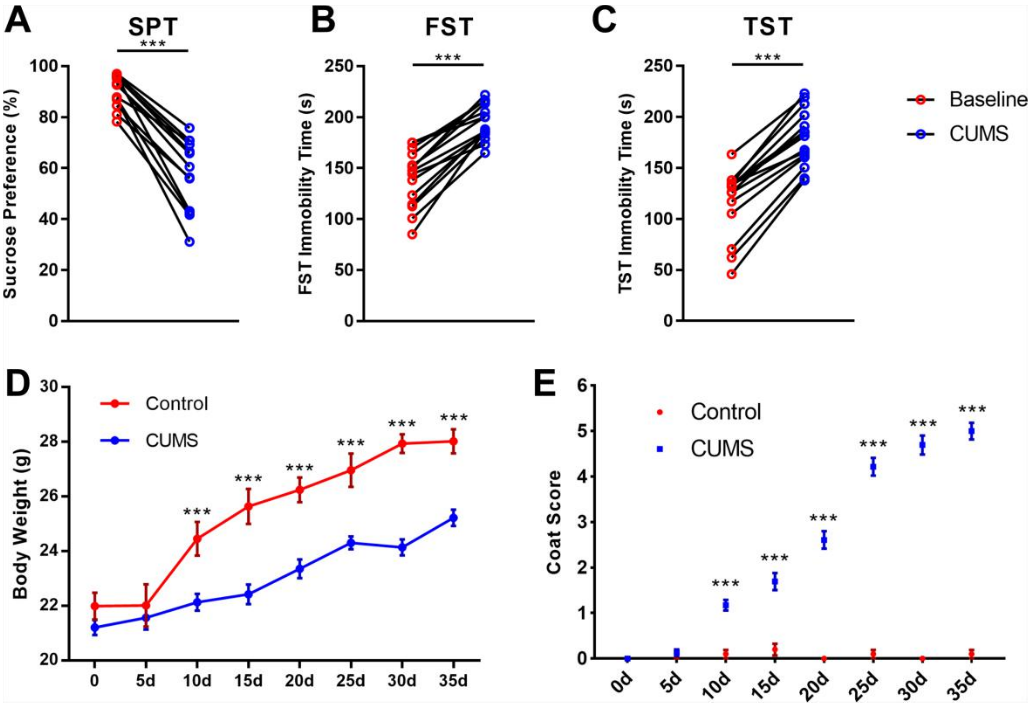

Figure S1. Establishment and assessment of depression-like phenotypes in mice, Related to Figure 2. (A) Results of the SPT in baseline and CUMS mice. **(B)** Duration of immobility in the FST in baseline and CUMS mice. (**C**) Duration of immobility in the TST in baseline and CUMS mice. n = 13**–**15 per group. Data points in **A–C** represent mean ± SEM. *** $p < 0.001$ versus baseline data using Student's t-test. (**D**) Body weight of age-matched control mice (n = 10) and CUMS mice (n = 23) at 35 days. Data are expressed as mean ± SEM. *** $p < 0.001$ versus control group using Student's t-test. (**E**) Coat scores of age-matched control mice (n = 10) and CUMS mice (n = 23) at 35 days. Data are expressed as mean ± SEM. *** $p < 0.001$ versus control group using Student's t-test. CUMS, chronic unpredictable mild stress; SPT, sucrose preference test; FST, forced swim test; TST, tail suspension test.

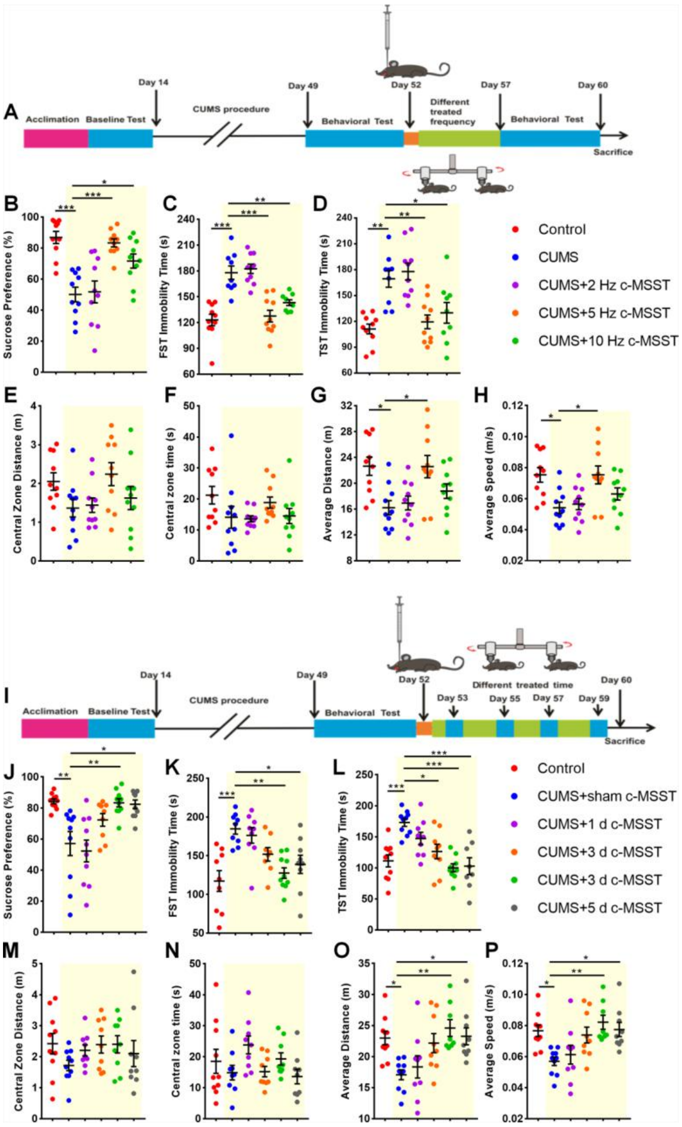

Figure S2. Selection and optimization of the therapeutic schedule of c-MSST in CUMS mice, Related to Figure 2. (**A**) Schematic diagram of the experimental procedure to determine the optimal frequency. (**B–D**) Both 5 Hz and 10 Hz c-MSST resulted in rapid antidepressant effects, as assessed by the SPT, FST and TST behavioural tests. (**E–H**) c-MSST had no significant effect on anxiety-like behaviours, but improvement in spontaneous locomotor activity during the OFT was observed in the group treated with 5 Hz c-MSST. (**I**) Schematic diagram of the experimental procedure for the optimal treatment duration with 5 Hz c-MSST. (**J–L**) As shown by the SPT and FST, 5 Hz c-MSST resulted in rapid antidepressant effects in CUMS mice after more than 5 days of treatment. TST abnormalities were rapidly ameliorated by 5 Hz c-MSST intervention for three days. (**M–P**) 5 Hz c-MSST has no significant effects on anxiety-like behaviours, but improvement in spontaneous locomotor activity was observed after treatment over 5 days. n = 8**–**10 mice per group. Data are expressed as mean ± SEM. * $p < 0.05$, ** $p < 0.01$, *** $p < 0.001$ using one-way ANOVA with Bonferroni correction. c-MSST, combined magnetic stimulation system treatment; CUMS, chronic unpredictable mild stress; SPT, sucrose preference test; FST, forced swim test; TST, tail suspension test; OFT, open field test.

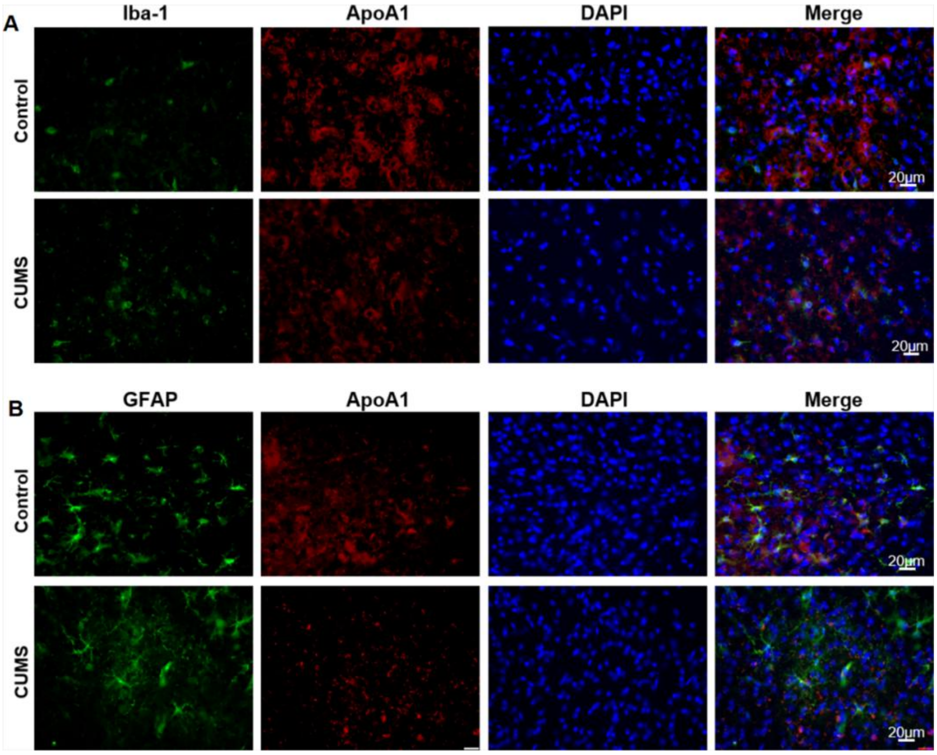

Figure S3. Cell-type-specific expression of ApoA1 in the left V1 region of mouse, Related to Figure 3. (**A and B)** Double immunofluorescence of ApoA1 (red) with iba-1 (green) or GFAP (green). Nuclei were stained blue with DAPI (blue). No typical double-labelled cells were detected. n=4 mice per group. Scale bars = 20 µm. ApoA1, apolipoprotein A; Iba-1, ionised calcium binding adaptor molecule 1; GFAP, glial fibrillary acidic protein; DAPI, 4',6-diamidino-2-phenylindole; CUMS, chronic unpredictable mild stress.

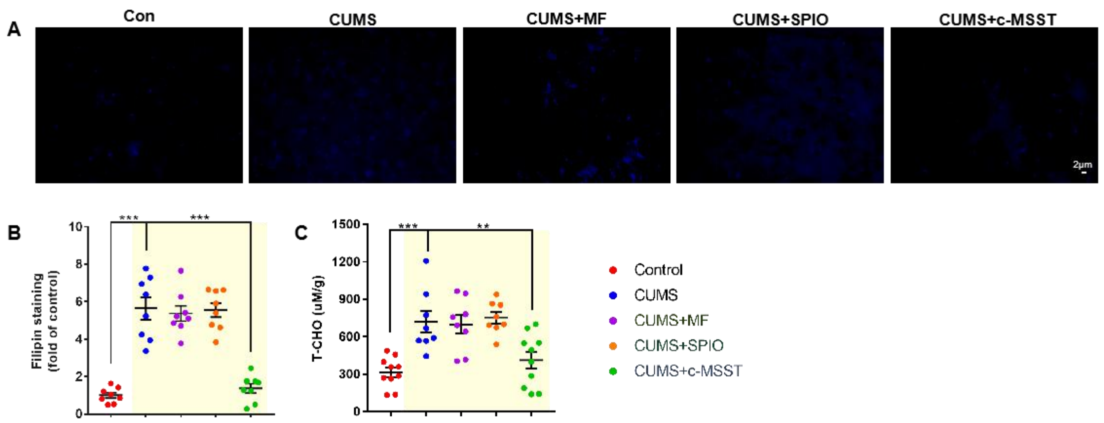

Figure S4. Measurement of cholesterol in different mouse groups, Related to Figure 3. (**A)** Filipin staining (blue) for cholesterol in mouse brain slices. (**B)** Quantification of Filipin staining (blue) in mouse brain slices. **C** Determination of cholesterol using an assay kit. n=8 mice per group. Data are expressed as the mean ± SEM. **p < 0.01, ***p < 0.001 using ANOVA with Bonferroni correction. CUMS, chronic unpredictable mild stress; MF, magnetic field; SPIO, superparamagnetic iron oxide; c-MSST, combined magnetic stimulation system treatment.

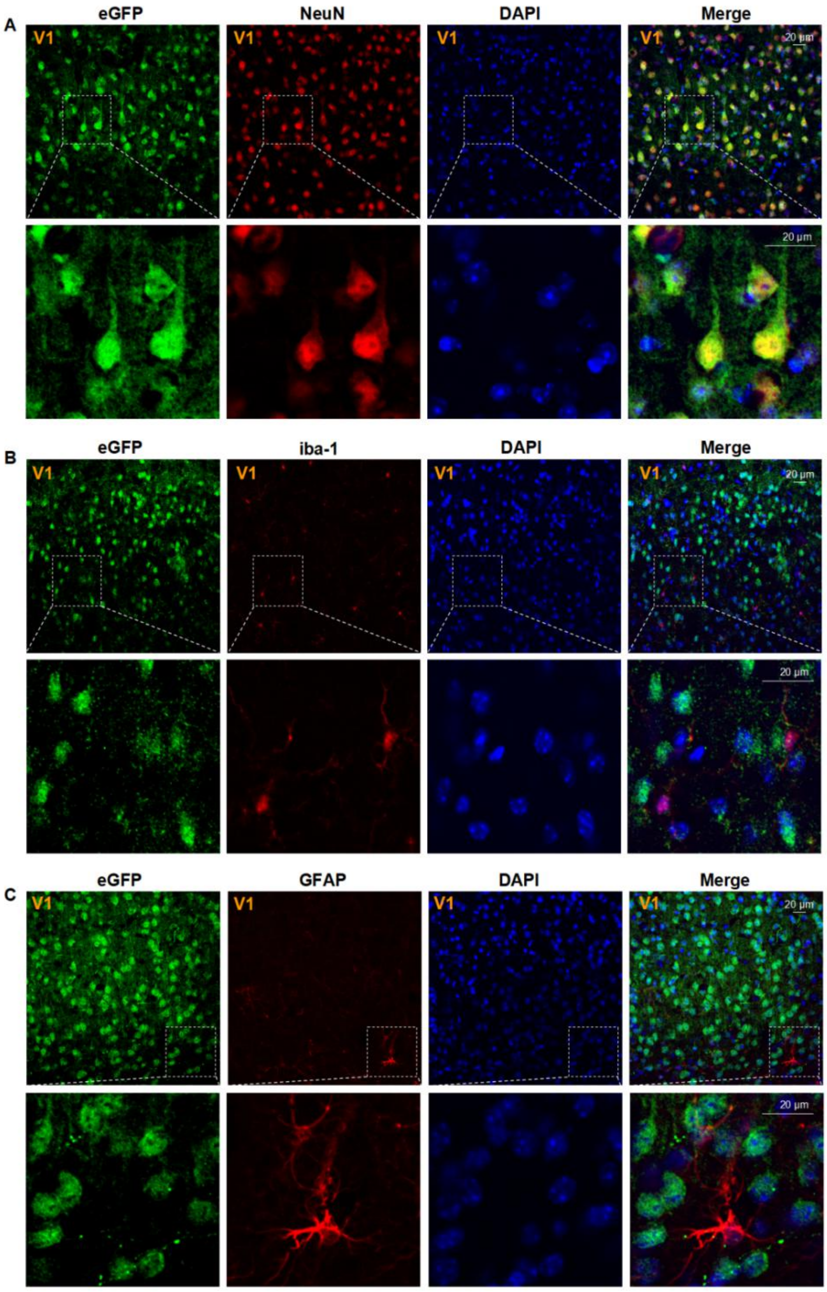

Figure S5. AAV only infect neurons, not non-neuronal cells in V1 of mice, Related to Figure 5. (A) Immunofluorescent double labelling for AAV (green)/NeuN (red), the lower image is an enlarged view of dashed boxed region in the upper image. (**B)** Immunofluorescent double labelling for AAV (green)/iba-1 (red), the lower image is an enlarged view of dashed boxed region in the upper image. (**C)** Immunofluorescent double labelling for AAV (green)/GFAP (red), the lower image is an enlarged view of dashed boxed region in the upper image. Nuclei were stained blue with DAPI. n=4 mice per group. Scale bar = 20 μm. eGFP, enhanced green fluorescent protein; NeuN, neuronal nuclei; iba-1, ionised calcium binding adaptor molecule 1; GFAP, glial fibrillary acidic protein; DAPI, 4',6-diamidino-2-phenylindole; AAV, adeno-associated virus.

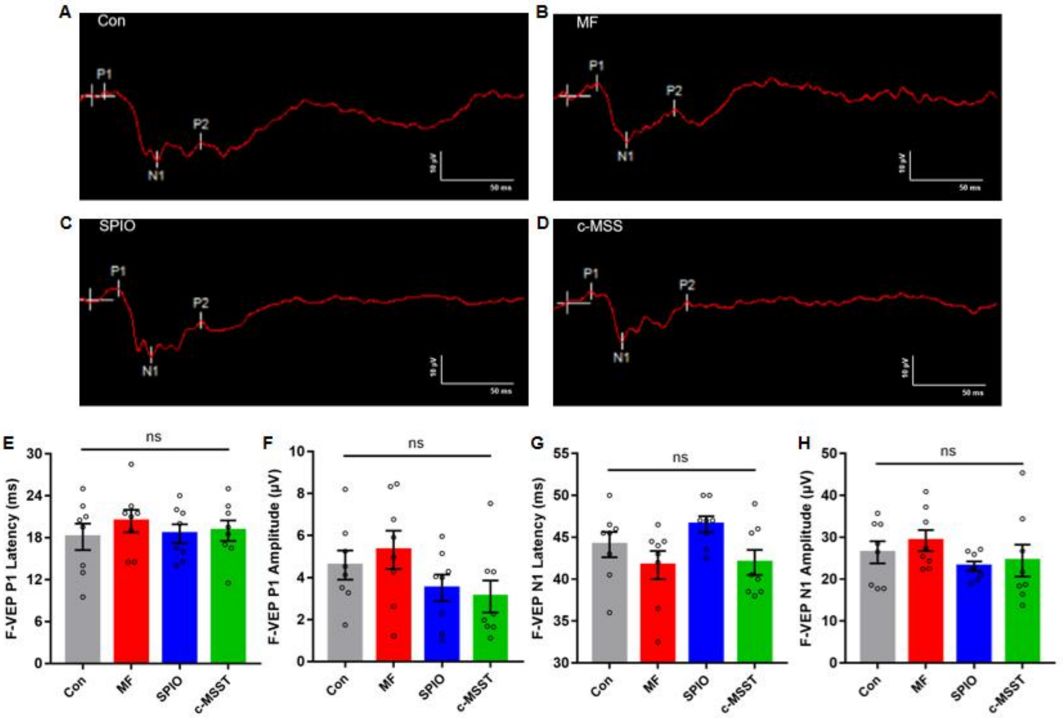

Figure S6. MF, SPIO and c-MSST condition does not affect visual function of mice, Related to Figure 7. (A-D) Representative F-VEP traces in Con, MF and c-MSS condition of mice. (**E and F)** No significant differences of the P1 latency and amplitude of the four groups of mice. n=8 mice per group. (**G and H)** No significant differences of the N1 latency and amplitude of the four groups of mice. n=8 mice per group. Data are expressed as mean ± SEM using one-way ANOVA with Bonferroni correction. ns, no significant difference; F-VEP, flash visual evoked potential; MF, magnetic field; SPIO, superparamagnetic iron oxide; c-MSST, combined magnetic stimulation system treatment.

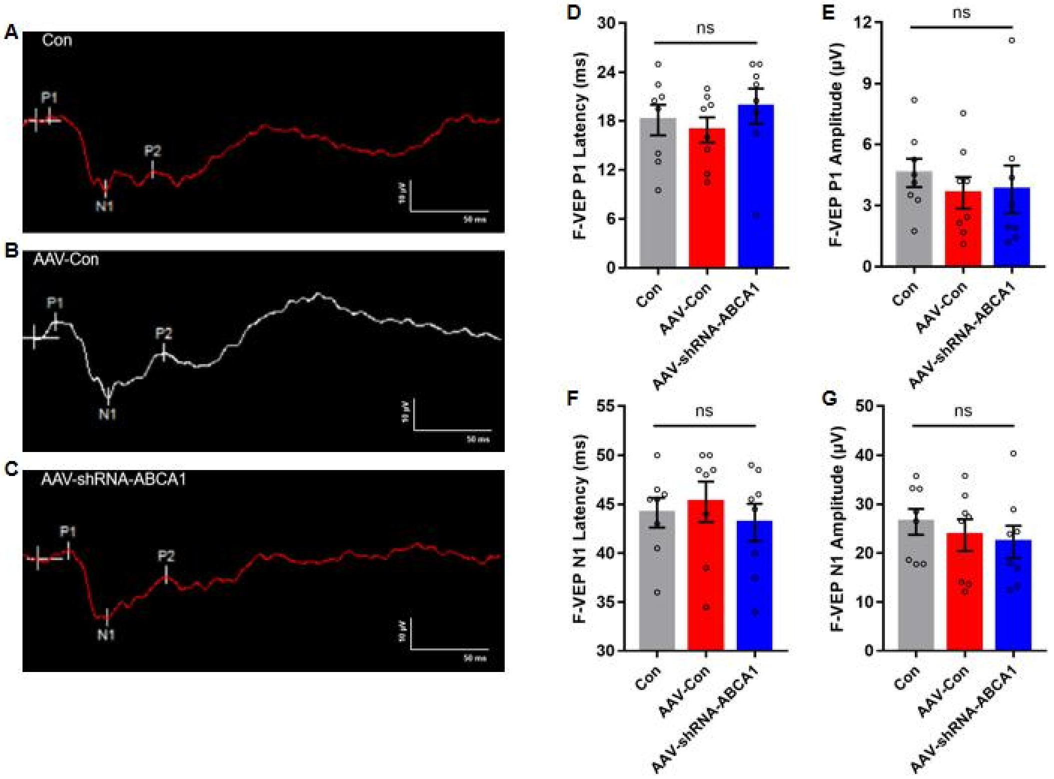

Figure S7. AAV condition does not affect visual function of mice, Related to Figure 7. (A-C) Representative F-VEP traces in Con, AAV-Con and AAV-shRNA-ABCA1 condition of mice. (**D and E)** No significant differences of the P1 latency and amplitude of the three mice groups. n=8 mice per group. (**F and G)** No significant differences of the N1 latency and amplitude of the three mice groups. n=8 mice per group. Data are expressed as mean ± SEM using one-way ANOVA with Bonferroni correction. ns, no significant difference; F-VEP, flash visual evoked potential; AAV, adeno-associated viral; ABCA1, ATP-binding cassette, subfamily A, member 1.

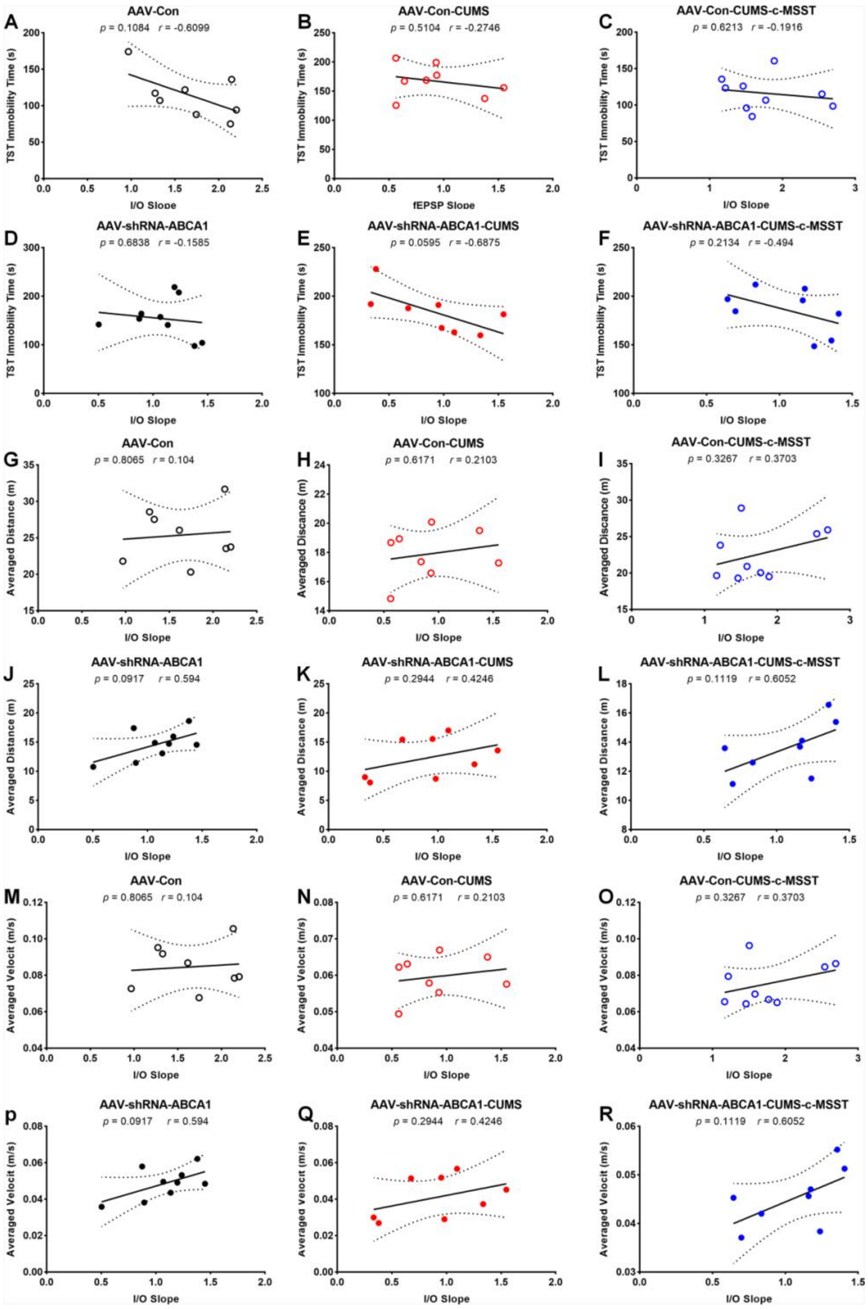

Figure S8. Correlation between the slope of I/O curves (basal synaptic transmission) and depression-like behaviors (TST and OFT) in different groups of mice measured by linear regression analysis and pearson's correlation (two-tailed), Related to Figure 8. AAV-Con group, black open circle; AAV-shRNA-ABCA1 group, black solid circle; AAV-Con + CUMS group, red open circle; AAV-shRNA-ABCA1 + CUMS group, red solid circle; AAV-Con + CUMS + c-MSST group, blue open circle; AAV-shRNA-ABCA1 + CUMS + c-MSST group, blue solid circle. (**A-F)** Correlation between the slope of I/O curves and TST in six different groups. (**G-R)** Correlation between the slope of I/O curves and OFT in six different groups. n = 8–9 mice per group. I/O, input/output; CUMS, chronic unpredictable mild stress; c-MSST, combined magnetic stimulation system treatment; TST, tail suspension test; OFT, open field test.

Table S1. Demographic and clinical characteristics at baseline in healthy controls and MDD patients, Related to Figure 4.

	HC (N = 55)	MDD (N = 74)	p Value
Age, years	34.04 ± 1.45	31.54 ± 1.39	0.225
Female, n (%)	34 (60.71%)	42 (56.76%)	0.563
Education, years	13.44 ± 0.60	12.31 ± 0.41	0.114
Family history, n (%)	1 (1.8%)	26 (35.14%)	0.000
First episode, n (%)	52 (70.27%)	NA	
Age of onset, years	27.53 ± 1.31	NA	
Disease duration (months)	49.46 ± 8.42	NA	
Smoking, n (%)	13 (23.64%)	13 (17.57%)	0.395
Drinking, n (%)	7 (12.73%)	7 (9.46%)	0.555
Baseline HAMD-24 scores	1.89 ± 0.30	35.12 ± 0.91	0.000

Abbreviations: HC, Healthy Control; MDD, Major Depressive Disorder; HAMD-24, 24-item Hamilton Depression Scale; NA, Not Available. Values are expressed as Mean ± SEM.

Table S2. The neuropsychological assessment scores from baseline to after 5 days of rTMS treatment between the real and sham subgroups, Related to Figure 4.

	Real r-TMS (N=45)		Sham r-TMS (N=26)	
	Day 0	Day 5	Day 0	Day 5
HAMD-24	35.33 ± 0.95	18.53 ± 1.44	34.58 ± 1.93	20.54 ± 2.29
HAMA	28.11 ± 1.12	14.33 ± 1.36	25.54 ± 2.06	14.19 ± 1.9
SDS	70.96 ± 1.48	55.58 ± 2.54	69.58 ± 2.7	53.81 ± 2.88
SAS	62.98 ± 1.67	50.49 ± 2.20	63.04 ± 3.14	49.92 ± 13.12
BHS	11.36 ± 0.59	7.69 ± 0.82	10.19 ± 1.16	7.54 ± 2.65
LES	44.47 ± 5.82	NA	48.31 ± 6.19	NA
CTQ-SF	51.49 ± 1.57	NA	52.65 ± 2.48	NA
APGAR	5.51 ± 0.47	NA	4.85 ± 0.58	NA
SCSR	43.33 ± 1.39	NA	46.38 ± 1.83	NA
Suicide Factor	2.18 ± 0.17	0.31 ± 0.10	2.08 ± 0.25	0.73 ± 0.18
Anxiety Factor	7.91 ± 0.27	4.22 ± 0.36	8.04 ± 0.53	4.54 ± 0.51
Weight Factor	0.71 ± 0.13	0.24 ± 0.09	0.77 ± 0.18	0.08 ± 0.05
Cognitive Disturbance Factor	7.40 ± 0.48	2.82 ± 0.44	7.04 ± 0.74	3.58 ± 0.63
Diurnal Variation Factor	0.89 ± 0.14	0.42 ± 0.10	0.81 ± 0.18	0.31 ± 0.12
Retardation Factor	8.98 ± 0.27	4.86 ± 0.40	9.12 ± 0.41	6.31 ± 0.64
Sleep Disturbance Factor	4.04 ± 0.28	2.60 ± 0.27	3.96 ± 0.42	2.54 ± 0.42
Hopelessness Factor	5.40 ± 0.27	3.31 ± 0.31	4.85 ± 0.36	3.31 ± 0.42

Abbreviations: rTMS, repetitive transcranial magnetic stimulation; HAMD-24: 24-item Hamilton Depression Scale; HAMA, Hamilton Anxiety Scale; SDS, Self-Rating Depression Scale; SAS, Self-Rating Anxiety Scale; BHS, Beck Hopelessness Scale; LES, Life Event Scale; CTQ-SF, Childhood Trauma Questionnaire-Short Form; APGAR, Adaptation Partnership Growth Affection Resolve; SCSR, Self-Consciousness Scale Revised; NA, Not Available. Values are expressed as Mean ± SEM.
